# Podocyte Regeneration Driven by Renal Progenitors Determines Glomerular Disease Remission and Can Be Pharmacologically Enhanced

**DOI:** 10.1016/j.stemcr.2015.07.003

**Published:** 2015-07-30

**Authors:** Laura Lasagni, Maria Lucia Angelotti, Elisa Ronconi, Duccio Lombardi, Sara Nardi, Anna Peired, Francesca Becherucci, Benedetta Mazzinghi, Alessandro Sisti, Simone Romoli, Alexa Burger, Beat Schaefer, Annamaria Buccoliero, Elena Lazzeri, Paola Romagnani

**Affiliations:** 1 Excellence Centre for Research, Transfer and High Education for the Development of DE NOVO Therapies (DENOTHE), University of Florence, Viale Pieraccini 6, 50139 Florence, Italy; 2 Department of Clinical and Experimental Biomedical Sciences, University of Florence, Viale Morgagni 50, 50134 Florence, Italy; 3 Nephrology Unit, Meyer Children’s University Hospital, Viale Pieraccini 24, 50141 Florence, Italy; 4 Institute of Molecular Life Sciences, University of Zurich, Winterthurerstrasse 190, 8057 Zürich, Switzerland; 5 Department of Oncology and Children’s Research Center, University Children’s Hospital, Steinwiesenstrasse 75, 8032 Zurich, Switzerland; 6 Pathology Unit, Meyer Children’s Hospital, Viale Pieraccini 24, 50141 Florence, Italy

## Abstract

Podocyte loss is a general mechanism of glomerular dysfunction that initiates and drives the progression of chronic kidney disease, which affects 10% of the world population. Here, we evaluate whether the regenerative response to podocyte injury influences chronic kidney disease outcome. In models of focal segmental glomerulosclerosis performed in inducible transgenic mice where podocytes are tagged, remission or progression of disease was determined by the amount of regenerated podocytes. When the same model was established in inducible transgenic mice where renal progenitors are tagged, the disease remitted if renal progenitors successfully differentiated into podocytes, while it persisted if differentiation was ineffective, resulting in glomerulosclerosis. Treatment with BIO, a GSK3s inhibitor, significantly increased disease remission by enhancing renal progenitor sensitivity to the differentiation effect of endogenous retinoic acid. These results establish renal progenitors as critical determinants of glomerular disease outcome and a pharmacological enhancement of their differentiation as a possible therapeutic strategy.

## Highlights

• Renal progenitors can generate differentiated podocytes after glomerular injury
• Renal progenitor response critically determines glomerular disease remission
• Inefficient renal progenitor to podocyte differentiation dictates disease chronicization
• Renal progenitor differentiation into podocytes can be enhanced with drugs

## Graphical Abstract

**Figure:**
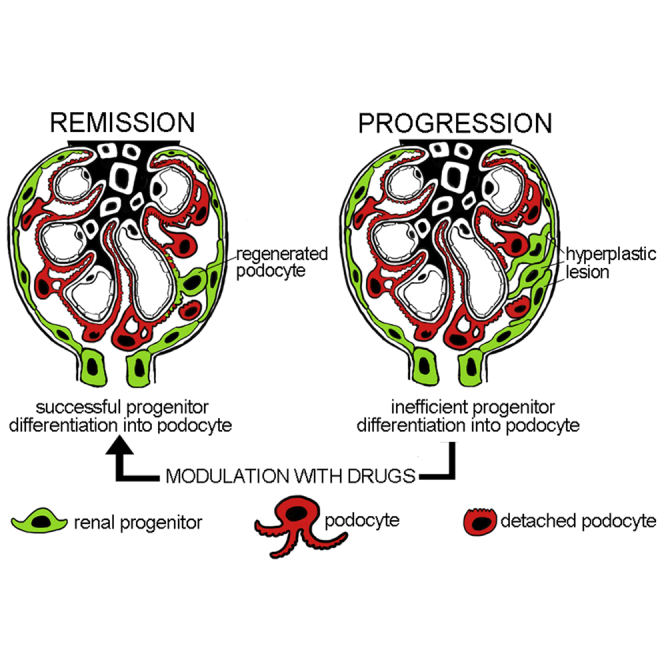

## Teaser

In this article, Romagnani, Lasagni, and colleagues show that podocyte regeneration is a critical determinant of the outcome of glomerular disorders and that enhancement of renal progenitor differentiation into podocyte through pharmacological treatment shifts the disease from progression to remission.

## Introduction

Chronic kidney diseases (CKDs) affects ∼10% of the population and represent a major global health burden (Eckardt et al., 2013). Worldwide, the number of patients with end-stage renal disease (ESRD) receiving renal replacement therapy is estimated at more than 1.4 million, with an annual growth rate of 8% (Schieppati and Remuzzi, 2005). ESRD represents only the tip of the iceberg; even early-stage CKD is associated with increased prevalence of numerous disorders and an increased risk of death.

The course of CKD can be extremely variable. Certain renal diseases quickly lead to irreversible ESRD. More common nephropathies do progress less rapidly but still evolve to ESRD at different rates or can show remission and even regression, spontaneously or following specific treatments. Even if pathophysiologic mechanisms of CKD progression are shared and independent of etiology, the reasons for this extreme outcome variability, even in patients affected by the same disorder, remain mostly unknown.

The majority of CKD originates from the glomerulus, where the podocyte, a highly differentiated cell representing the main constituent of the filtration barrier, is the culprit. Indeed, dysfunction and loss of glomerular podocytes are the driving forces for CKD initiation and progression (Kriz and LeHir, 2005; Chen and Miner, 2012; Wiggins, 2007). Clinically, this is evidenced by proteinuria, and unfortunately, there are no clinical methods to repair podocyte damage. Podocytes are post-mitotic cells that typically do not divide but can undergo hypertrophy in an attempt to cover the underlying glomerular basement membrane in exposed areas where neighboring cells have detached or died (Wanner et al., 2014). However, if the injury exceeds a certain threshold, podocyte hypertrophy reveals itself to be an unfit strategy over time, as the loss of podocytes and segmental sclerosis lead to podocyte detachment and a decreased ultrafiltration capacity (Wiggins, 2007; Lasagni et al., 2013). Therefore, the identification of effective ways to promote podocyte regeneration has become a major focus of research.

In recent years, some findings have suggested that renal progenitor cells (RPCs) may exist in humans and represent a potential source for podocyte replacement (Romagnani, 2009; Romagnani et al., 2013; Shankland et al., 2014). In humans, RPCs represent a subset of parietal epithelial cells (PECs) in the Bowman’s capsule that exhibit functional progenitor features and are characterized by co-expression of two species-specific markers, CD133 and CD24 (Romagnani et al., 2013; Shankland et al., 2014; Sagrinati et al., 2006; Ronconi et al., 2009). Studies using mouse models report the capacity of PECs to differentiate into podocytes, but only during kidney development (Berger et al., 2014; Appel et al., 2009). Rather, in certain conditions, PEC activation can be harmful and drives generation of hyperplastic intraglomerular cellular lesions (Smeets et al., 2009), leading to nephron degeneration (Kriz and LeHir, 2005).

In this study, we hypothesized that the response of RPCs to podocyte injury may determine the outcome of glomerular disorders and that enhancement of podocyte regeneration provided by RPCs may represent a new target for the treatment of CKD.

## Results

### Remission of Glomerular Disease after Podocyte Injury Is Associated with the Generation of Novel Podocytes

**Figure 1:**
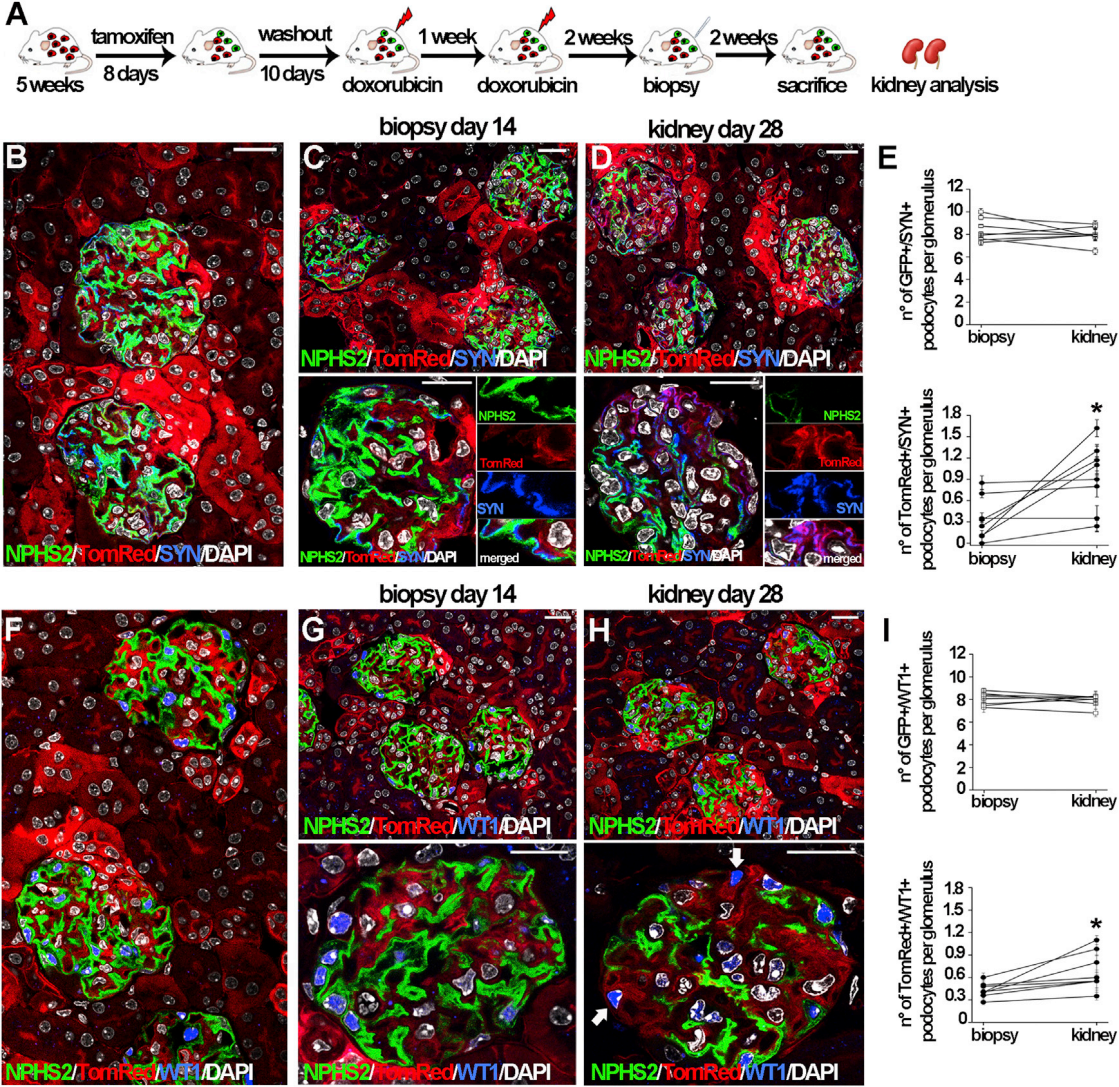
Podocyte Regeneration Occurs after Podocyte Detachment and Is Not Driven by Mitosis of Pre-existing Podocytes (A) Experimental scheme. (B) Healthy kidney from a *NPHS2.iCreERT2;mT/mG* mouse stained with anti-SYN (blue). (C and D) Low (top) and high (bottom) magnification of a renal biopsy specimen from a doxorubicin-treated *NPHS2.iCreERT2;mT/mG* mouse at day 14 (C) and the kidney of the same mouse at day 28 (D) stained with anti-SYN (blue). (E) Number of pre-existing podocytes (detected as green and blue cells, GFP+/SYN+) (top) and number of newly generated podocytes (detected as red and blue cells, TomRed+/SYN+) (bottom) per glomerular section of all glomeruli in the biopsy specimen (day 14) and kidney (day 28) (n = 8) of each mouse; ^∗^p < 0.05 (day 28) by Wilcoxon test. (F) Healthy kidney from a *NPHS2.iCreERT2;mT/mG* mouse stained with anti-WT1 (blue). (G and H) Low (top) and high (bottom) magnification of renal biopsy from a doxorubicin-treated *NPHS2.iCreERT2;mT/mG* mouse at day 14 (G) and kidney of the same mouse at day 28 (H) stained with anti-WT1 (blue). (I) Number of pre-existing podocytes (detected as green and blue cells, GFP+/WT1+) (top) and number of newly generated podocytes (detected as red and blue cells, TomRed+/WT1+) (bottom) per glomerular section of all glomeruli in the biopsy specimen (day 14) and kidney (day 28) (n = 8) of each mouse; ^∗^p < 0.05 (day 28) by Wilcoxon test. Data represent the mean ± SEM; DAPI counterstains nuclei. Scale bar, 20 μm.

To test whether the generation of new podocytes after injury can influence disease outcome, we first used *NPHS2.iCreER*^*T2*^*;mT/mG* mice. In this inducible transgenic model, following tamoxifen administration (Figure 1A), membrane-targeted GFP genetically labels NPHS2-expressing cells (podocytes) green, while all the other kidney cells are labeled red with TomatoRed (TomRed). However, after tamoxifen withdrawal, newly generated podocytes will also be labeled red and can be identified as red cells that are co-stained with anti-synaptopodin (anti-SYN) or anti-WT1 antibodies. In healthy mice, 8 days of tamoxifen administration followed by a washout period of 10 days resulted in near 100% efficiency and specificity of GFP expression (96.27% ± 0.64%), as demonstrated by the virtually complete overlap between GFP-labeled podocytes and podocytes labeled with an anti-SYN blue-conjugated antibody (Figure 1B). After tamoxifen washout, *NPHS2.iCreER*^*T2*^*;mT/mG* mice received two doxorubicin injections with a 1-week break in between (Figure 1A) to induce Adriamycin nephropathy, a model of human focal segmental glomerulosclerosis (FSGS) (Lee and Harris, 2011), in C57Bl/6 mice (Peired et al., 2013). To evaluate the occurrence of podocyte regeneration at the single-mouse level, avoiding the influence of interindividual variability in the efficiency of Cre recombinase activation, we performed serial biopsies at day 14 from the second doxorubicin injection in a group of eight animals and counted the number of GFP+/SYN+ cells (pre-existing podocytes) and the number of TomRed+/SYN+ cells (newly generated podocytes) in glomeruli at days 14 and 28 from the second doxorubicin injection in each mouse. Representative images of glomeruli in the biopsy specimen and kidney of the same mouse are shown in Figures 1C and 1D. A significant increase of TomRed+/SYN+ newly generated podocytes was observed (Figure 1E). Similar results were obtained when the same biopsies were stained with anti-WT1 antibody and cells counted as GFP+/WT1+ cells (pre-existing podocytes) or TomRed+/WT1+ cells (newly generated podocytes; Figures 1F–1I).

**Figure 2:**
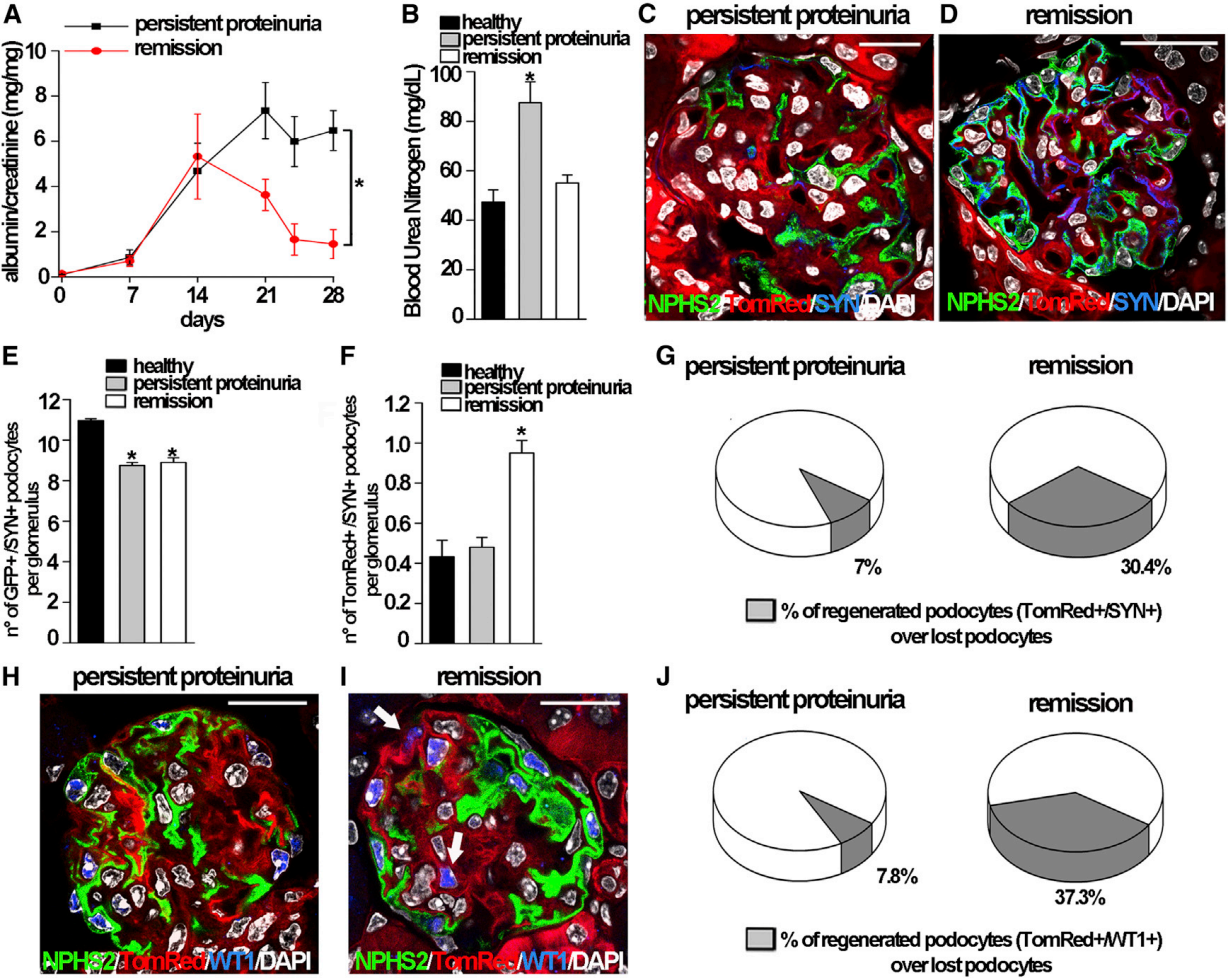
Remission of Glomerular Disease in Mice with Adriamycin Nephropathy Is Associated with the Generation of Novel Podocytes (A) Albumin/creatinine levels in persistent proteinuric *NPHS2.iCreERT2;mT/mG* mice (n = 10) and mice undergoing remission (n = 5). Day 0 corresponds to the time of second doxorubicin injection; ^∗^p < 0.05 (day 28) by Mann-Whitney test. (B) Blood urea nitrogen levels in healthy (n = 4), progressed (n = 10), and remitted (n = 5) mice at day 28; ^∗^p < 0.05 versus healthy mice and mice with proteinuria remission by Mann-Whitney test. (C and D) Representative kidney of a *NPHS2.iCreERT2;mT/mG* mouse with persistent proteinuria (C) and of a *NPHS2.iCreERT2;mT/mG* mouse with proteinuria remission (D) stained with anti-SYN (blue). (E) Number of pre-existing podocytes (detected as green and blue cells, GFP+/SYN+) per glomerular section of all glomeruli in healthy (n = 4) and persistently proteinuric mice (n = 10) and in mice with proteinuria remission (n = 5) at day 28. ^∗^p < 0.05 versus healthy mice by Mann-Whitney test. (F) Number of newly generated podocytes (detected as red and blue cells, TomRed+/SYN+) per glomerular section of all glomeruli in healthy (n = 4) and persistently proteinuric mice (n = 10) and in mice with proteinuria remission (n = 5) at day 28. ^∗^p < 0.05 versus healthy and persistently proteinuric mice by Mann-Whitney test. (G) Percentage of regenerated podocytes over lost podocytes (n = 10 for proteinuric mice and n = 5 for mice with proteinuria remission). (H and I) Representative kidney of a *NPHS2.iCreERT2;mT/mG* mouse with persistent proteinuria (H) and of a *NPHS2.iCreERT2;mT/mG* mouse with proteinuria remission (I) stained with anti-WT1 (blue). (J) Percentage of regenerated podocytes over lost podocytes (n = 10 for proteinuric mice and n = 5 for mice with proteinuria remission). Data represent the mean ± SEM; DAPI counterstains nuclei. Scale bar, 20 μm.

To further evaluate whether podocyte regeneration is associated with proteinuria remission, we induced Adriamycin nephropathy in *NPHS2.iCreER*^*T2*^*;mT/mG* mice and monitored the urinary albumin/creatinine ratio for 28 days, starting from the second doxorubicin injection (day 0). At day 28, on the basis of the proteinuria course, we divided the mice with persistent proteinuria (increase or persistence of proteinuria in the nephrotic range at day 28) from animals that underwent proteinuria remission (reduction of proteinuria of at least 50% of the peak values at day 28), based on the KDIGO Guidelines (Figure 2A) (KDIGO, 2012). In mice with persistently nephrotic proteinuria, we observed a statistically significant increase in blood urea nitrogen (BUN) levels at day 28 in comparison to both healthy mice and mice with proteinuria remission (Figure 2B). At day 28, we analyzed kidneys by confocal microscopy and counted the number of GFP+/SYN+ cells and the number of TomRed+/SYN+ cells in glomeruli of healthy mice, mice with persistent proteinuria and CKD, and mice that underwent proteinuria remission. Representative images of glomeruli of persistently proteinuric mice and mice with proteinuria remission stained with an anti-SYN antibody are shown in Figures 2C and 2D, respectively. In mice with persistently nephrotic proteinuria, we counted 8.75 ± 0.15 GFP+/SYN+ podocytes and 0.48 ± 0.05 TomRed+/SYN+ podocytes per glomerular section. By contrast, in mice with proteinuria remission, we observed 8.9 ± 0.23 GFP+/SYN+ podocytes and 0.95 ± 0.06 TomRed+/SYN+ podocytes per glomerular section (Figures 2E and 2F). Only the number of TomRed+/SYN+ podocytes was significantly different in mice with persistent proteinuria versus mice with proteinuria remission, suggesting that the different podocyte number observed at day 28 in these groups of mice was related to a different amount of podocyte regeneration. Indeed, once we subtracted the background of TomRed+/SYN+ cells over total podocytes present in healthy kidney, the percentage of newly generated podocytes was 1.43% in mice with persistent proteinuria and 5.9% in mice with proteinuria remission when calculated over the total amount of kidney podocytes. However, since podocyte injury is focal and segmental and only a limited amount of podocytes is lost, this corresponds to regeneration of 30.4% of lost podocytes in mice with proteinuria remission versus only 7.0% in mice with persistent proteinuria (Figure 2G; see Supplemental Experimental Procedures for a detailed description of the calculation). Similar results were obtained when the kidney tissues were stained with an anti-WT1 antibody (Figures 2H–2J).

Taken together, these results demonstrate that podocyte regeneration is associated with proteinuria remission, while lack of podocyte regeneration is associated with persistent nephrotic proteinuria and development of CKD. These results also suggest that newly generated podocytes are derived not from already existing podocytes but rather from an unknown podocyte progenitor.

### PAX2+ Cells Represent Progenitors that Generate Novel Podocytes during Kidney Growth

**Figure 3:**
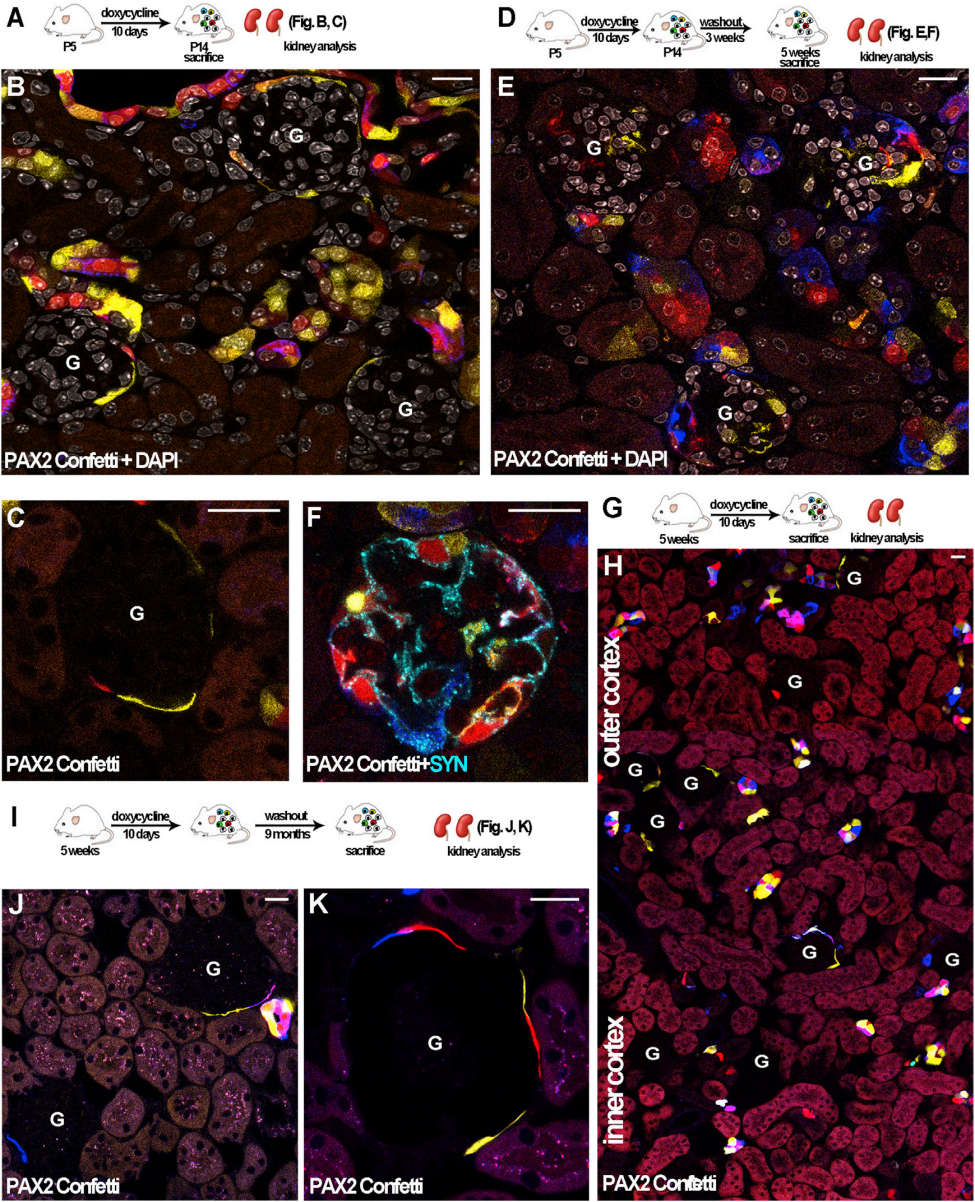
PAX2+ Cells Located in Parietal Epithelium of the Bowman’s Capsule Are Progenitors for Podocytes during Postnatal Kidney Growth (A) Experimental scheme for results shown in (B) and (C). (B) Kidney of *Pax2.rtTA;TetO.Cre;R26.Confetti* mice induced at P5 for 10 days and sacrificed at 2 weeks of age (n = 8). Multicolor Confetti tracing is shown in red, yellow, and cyan. PAX2+ progenitors are located in parietal epithelium of the Bowman’s capsule and in tubular structures. (C) Higher magnification of a glomerulus showing PAX2+ cells only in parietal epithelium of the Bowman’s capsule. (D) Experimental scheme for results shown in E and F. (E) Kidney of *Pax2.rtTA;TetO.Cre;R26.Confetti* mice induced at P5 for 10 days when doxycycline was suspended and mice tracked until 5 weeks of age (n = 8). Fluorescent cells were present in parietal epithelium of the Bowman’s capsule as well as inside the glomeruli. (F) Higher magnification of a glomerulus showing fluorescently labeled PAX2+ cells inside the tuft, positively stained with anti-SYN antibody (light blue), demonstrating their differentiation into podocytes. (G) Experimental scheme for results shown in (H). (H) Juxtaposed confocal images from top (outer cortex) to bottom (inner cortex) of an adult *Pax2.rtTA;TetO.Cre;R26.Confetti* mouse kidney. (I) Experimental scheme for results shown in (J) and (K). (J) Glomeruli with PAX2+ capsule in mice induced at 5 weeks of age for 10 days, then doxycycline was suspended and mice tracked for the following 9 months (n = 6). (K) Higher magnification of a glomerulus with PAX2+ cells in the Bowman’s capsule. G, glomerulus. DAPI counterstains nuclei (white). Scale bar, 20 μm. See also Figure S1.

To establish a model for RPC lineage tracing, we decided to make use of the regulatory region of the transcription factor PAX2, a critical regulator of the embryonic progenitor population during kidney development in vertebrates (Dressler, 2008). PAX2 is co-expressed with CD133 in RPCs of the adult human kidney (Bussolati et al., 2005; Ye et al., 2011) and may thus be ideal for lineage tracing of RPCs of the Bowman’s capsule in the mouse. A transgenic *Pax2-rtTA* mouse strain obtained through genetic recombineering using a large bacterial artificial chromosome (BAC) clone that drives expression of TetO-controlled transgenes upon doxycycline treatment in natively PAX2-expressing tissues was previously described and fully validated (Burger et al., 2011). This mouse was bred with the Confetti reporter to combine inducible lineage tracing of PAX2+ cells with clonal analysis (*Pax2.rtTA;TetO.Cre;R26.Confetti*; Figure S1A). Indeed, in this reporter, green, yellow, cyan, or red fluorescent protein is randomly expressed under the control of the *Pax2* promoter, allowing the tracing of single PAX2-expressing cells and of their progeny. Since PAX2 can also be expressed by podocyte precursors during kidney development but is lost upon their differentiation into mature podocytes and remains only in the PECs of the Bowman’s capsule in the post-natal kidney (Ryan et al., 1995), in a preliminary series of experiments, we induced pups with doxycycline through breastfeeding at different time points and for variable periods of time after birth. These experiments showed that when doxycyline induction was started at post-natal day 5 (P5) and went on for 10 days, ending at P14 (Figure 3A), PAX2 was expressed only by scattered cells within tubular structures as well as by PECs of the Bowman’s capsule (Figure 3B), while podocytes were not labeled (Figure 3C). At P5, the generation of new glomeruli from the metanephric mesenchyme (MM) has already ended (Short et al., 2014), but glomerular shaping up to stage V can continue until P10 (Kazimierczak, 1980). However, when doxycyline is administered by breastfeeding, at least 5 days of treatment are needed before Cre recombination occurs in this model, and during this phase, PAX2 expression in podocytes is shut off, thus explaining the selectivity of PAX2 labeling to PECs of the Bowman’s capsule at P14. Since nephron development has already ended at P14 (Hartman et al., 2007), this represents the optimal starting point to determine whether PAX2+ PECs contribute to post-natal glomerular growth. To this aim, in a parallel group of mice, doxycycline induction was started at P5, and after 10 days of induction, doxycycline was removed at P14, and mice were chased until the fifth week of age and then analyzed (Figure 3D). While at P14 virtually no podocytes were labeled, after chasing up to the fifth week of age, labeled cells were observed not only as PECs of the Bowman’s capsule but also inside the glomeruli (0.04 ± 0.03 at P14 versus 0.9 ± 0.16 after chasing up to 5 weeks; p < 0.05; Figure 3E). These intraglomerular PAX2-derived cells expressed SYN (Figure 3F), demonstrating their podocyte nature. When we removed doxycycline and started chasing experiments, only PECs of the Bowman’s capsule exhibited PAX2 labeling within glomeruli. This excludes the possibility that the labeled podocytes observed at 5 weeks were generated during development and demonstrates that they could only be derived from the PAX2+ PECs present at P14. Since 11.2 ± 0.5 podocytes are present on average in each glomerular section of an adult mouse at 5 weeks of age, this suggests that ∼8% of podocytes in adult glomeruli are recruited from PAX2+ cells during postnatal glomerular growth. These results demonstrate that PAX2+ cells localized within the Bowman’s capsule represent podocyte progenitors also once development has ended. Newly generated podocytes within glomeruli were labeled with different colors, suggesting they are not the result of clonal division of a single progenitor and are derived from migration and differentiation of multiple different progenitor cells within the same glomerulus (Figures 3E and 3F). To verify the persistence of PAX2+ progenitors in the adult mouse kidney, animals were then induced at 5 weeks of age for 10 days and then sacrificed (Figure 3G). No Cre recombinase activity was observed in absence of doxycycline treatment (Figure S1B). We observed PAX2+ cells localized as PECs in the Bowman’s capsule and scattered within the tubular structures (Figure 3H). Podocytes were not labeled. Interestingly, glomeruli with PAX2+ Bowman’s capsules were differently distributed between the different areas of the cortex (Figure 3H), with a higher presence of positive glomeruli in the outer cortex in comparison to the inner cortex (Figure 3H). On average, more than 50% of glomeruli in the outer cortex exhibited PAX2+ cells in the Bowman’s capsule. No further podocytes were recruited from PAX2+ progenitors from the fifth week up to 10 months of the life of the animal (Figures 3I–3K).

Taken together, these results demonstrate that *Pax2.rtTA;TetO.Cre;R26.Confetti* mice allow specific tracking of RPCs in the Bowman’s capsule of the mouse kidney.

### Remission of Glomerular Disease Depends upon Differentiation of PAX2 Progenitors into Novel Podocytes

**Figure 4:**
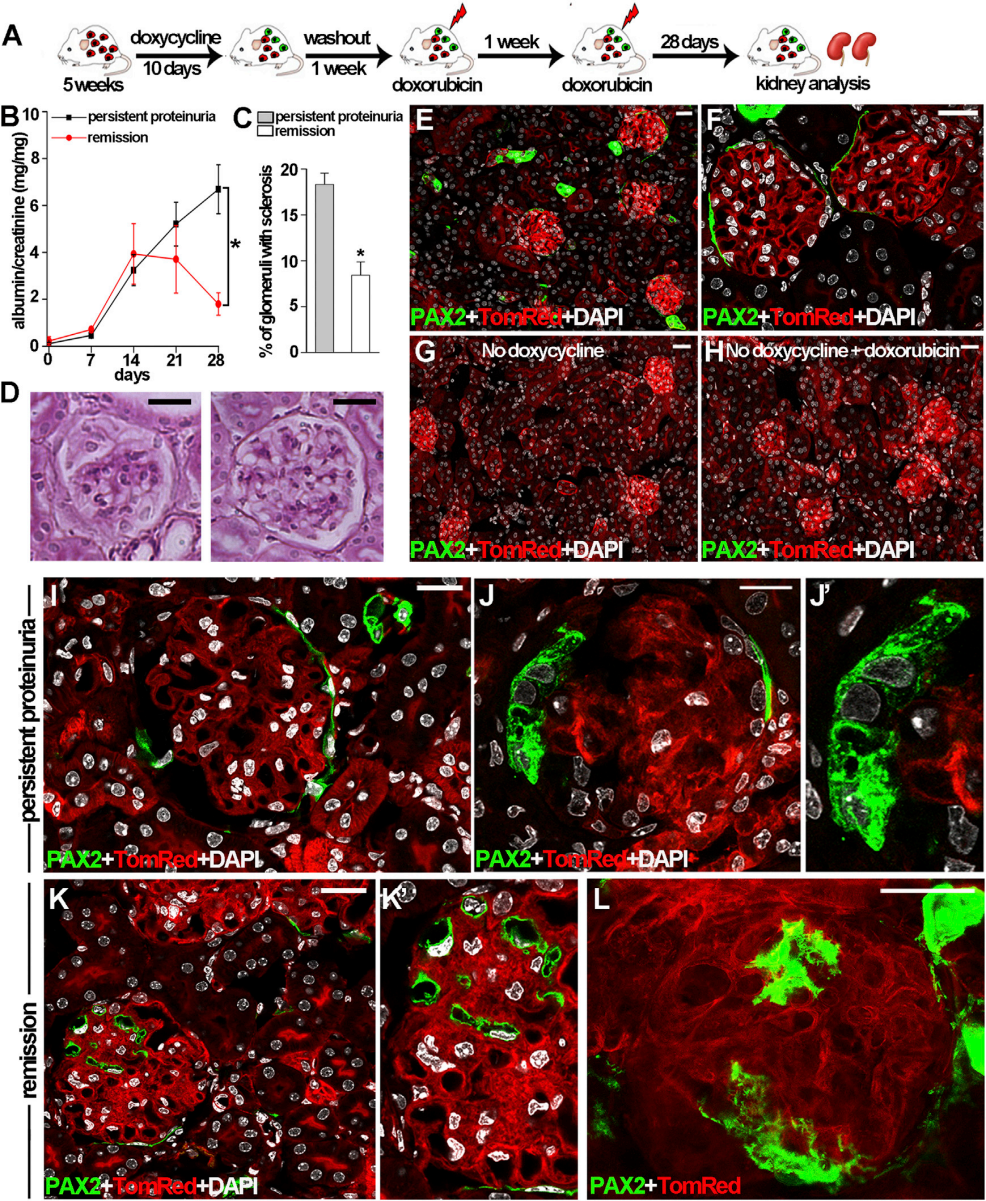
PAX2+ Progenitors Generate Lesions in Mice with Persistent Proteinuria and Podocytes in Animals with Proteinuria Remission (A) Experimental scheme. (B) Albumin/creatinine levels in persistent proteinuric *Pax2.rtTA;TetO.Cre;mT/mG* mice (n = 11) and mice undergoing remission (n = 5). Day 0 corresponds to the time of second doxorubicin injection; ^∗^p < 0.05 (day 28) by Mann-Whitney test. (C) Percentage of glomeruli with sclerosis in mice with persistent proteinuria (n = 11) and mice undergoing remission (n = 5). ^∗^p < 0.05 versus persistently proteinuric mice (Mann-Whitney test). (D) Representative periodic acid-Schiff staining of renal sections of persistently proteinuric mice (left) and of mice undergoing remission (right). (E and F) Representative glomeruli with PAX2+ capsule in healthy *Pax2.rtTA;TetO.Cre;mT/mG* mice induced at 5 weeks of age (n = 4). (G and H) Absence of basal Cre recombinase activity in the kidney of *Pax2.rtTA;TetO.Cre;mT/mG* healthy mice maintained with water without doxycycline and analyzed at 12 weeks of age (n = 3) (G) and in the kidney of *Pax2.rtTA;TetO.Cre;mT/mG* mice with Adriamycin nephropathy (n = 3) maintained with water without doxycycline and sacrificed 28 days after the second doxorubicin injection (H). (I) Representative kidney from a *Pax2.rtTA;TetO.Cre;mT/mG* mouse with persistent proteinuria. No green cells are present inside the glomeruli. (J) Representative kidney from a *Pax2.rtTA;TetO.Cre;mT/mG* mouse with persistently nephrotic proteinuria, showing PAX2+ cells in a hyperplastic lesion. (J′) Higher magnification of (J). (K) Representative kidney from a *Pax2.rtTA;TetO.Cre;mT/mG* mouse with proteinuria remission, where PAX2+ cells are observed inside the glomerular tuft. (K′) Higher magnification of (K). (L) 3D reconstruction of a glomerulus showing PAX2+ cells in epithelium of the Bowman’s capsule and PAX2-derived cells inside the glomerulus. Data represent the mean ± SEM. DAPI counterstains nuclei (white). Scale bar, 20 μm. See also Figure S2 and Movie S1.

To evaluate if PAX2+ progenitors could be responsible for the podocyte regeneration observed in the *NPHS2.iCreER*^*T2*^*;mT/mG* model, we induced Adriamycin nephropathy in *Pax2.rtTA;TetO.Cre;mT/mG* mice (Figure S2A) following the same protocol used for *NPHS2.iCreER*^*T2*^*;mT/mG* mice and shown in Figure 4A. We then monitored urinary albumin/creatinine ratio for 28 days and divided the mice with persistent proteinuria from those with proteinuria remission as described above (Figure 4B). At day 28, mice with persistent proteinuria had an albumin/creatinine ratio of 6.7 ± 1.03, whereas the ratio in mice with proteinuria remission was 1.8 ± 0.47 (p < 0.05); animals were sacrificed and kidneys analyzed. Consistently, mice with persistent proteinuria showed a higher percentage of glomeruli with sclerosis in comparison to mice with proteinuria remission (Figures 4C and 4D). In healthy mice, PAX2+ progenitors were localized exclusively in the PECs of the Bowman’s capsule (Figures 4E and 4F). In healthy mice of comparable age that were not treated with doxycycline, no green signal was observed, showing that there was no transgene leakage (Figure 4G). The green signal was also absent at day 28 after induction of Adriamycin nephropathy if doxycycline had not been administered, further demonstrating that this system had no leakage, even after doxorubicin treatment (Figure 4H). By contrast, in mice treated with doxycycline before induction of Adriamycin nephropathy, green fluorescent cells could be observed also inside glomeruli 28 days after injury (Figures 4I–4L). However, in mice with persistently nephrotic proteinuria, intraglomerular PAX2-derived green cells were virtually not detected (Figure 4I) or were observed as components of adhesions or of hyperplastic intraglomerular lesions (Figures 4J and 4J′). By contrast, in mice with proteinuria remission, intraglomerular PAX2+ cells were observed and localized around capillaries (Figures 4K, 4K′, and 4L) in virtually normal tufts, and z stack sectioning of glomeruli showed their continuity with PAX2+ PECs of the Bowman’s capsule (Figure S2B). Of note, these intraglomerular cells derived from PAX2+ PECs showed an arborized morphology (Figure 4L; Movie S1) suggestive of podocyte features.

**Figure 5:**
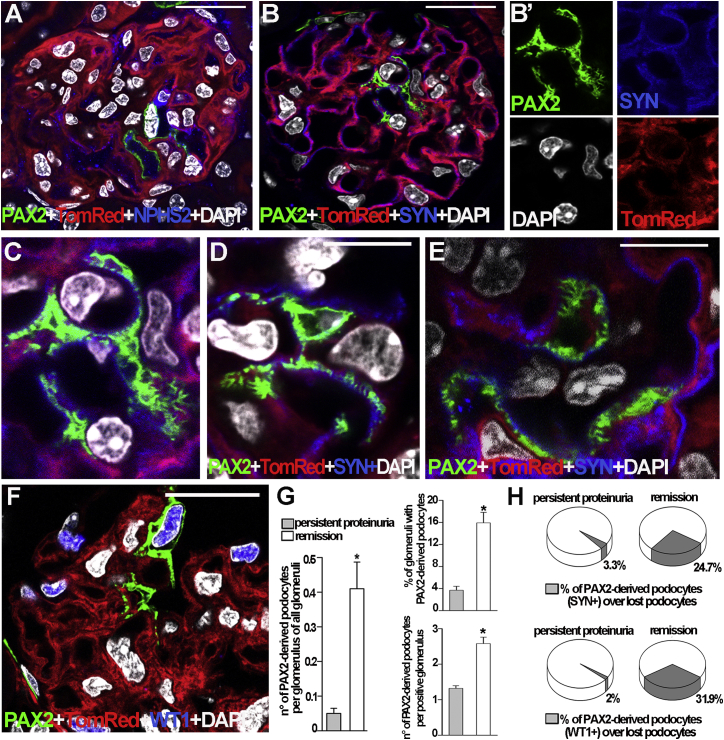
PAX2+ Progenitors Generate Fully Differentiated Podocytes in Mice with Proteinuria Remission (A and B) In *Pax2.rtTA;TetO.Cre;mT/mG* mouse with proteinuria remission, PAX2-derived cells observed inside the glomeruli expressed the podocyte marker podocin (NPHS2, blue) (A) and SYN (blue) (B). (B′) Split images of the PAX2+/SYN+ cell inside the glomerulus shown in (B). (C) Higher magnification of foot processes in the PAX2+/SYN+ cell shown in (B). (D and E) Higher magnification of PAX2+/SYN+ cells with foot processes. (F) Representative image of a PAX2-derived cell inside the glomerulus expressing the podocyte marker WT1 (blue). (G) Left. Quantification of PAX2+SYN+ cells/glomerular section of all glomeruli in mice with persistent proteinuria (n = 11) and mice undergoing remission (n = 5). ^∗^p < 0.05 versus persistently proteinuric mice (Mann-Whitney test). Right: percentage of glomeruli with PAX2-derived podocytes inside the glomerular tuft (top) and quantification of PAX2-derived podocytes/glomerular section of positive glomeruli (bottom). (H) Percentage of PAX2-derived podocyte over lost podocytes in animals with persistent proteinuria (n = 11) and mice with proteinuria remission (n = 5). Data represent the mean ± SEM. DAPI counterstains nuclei. Scale bar represents 20 μm, except in (D) and (E) (10 μm).

To analyze the phenotypic characteristics of PAX2+ cells observed within glomeruli of mice with proteinuria remission, we evaluated the expression of the podocyte markers podocin (NPHS2), SYN, and WT1. PAX2+ cells were intensely stained with anti-NPHS2 (Figure 5A), anti-SYN antibody (Figures 5B–5E), and anti-WT1 antibody (Figure 5F). Moreover, these cells showed characteristics of fully differentiated podocytes, presenting foot processes indistinguishable from resident podocytes (Figures 5C–5F).

Overall, we counted 0.41 ± 0.07 PAX2+/SYN+ cells per glomerular section in *Pax2.rtTA;TetO.Cre;mT/mG* mice with proteinuria remission, which corresponded to the regeneration of 24.7% of lost podocytes (Figures 5G and 5H). By contrast, in mice with persistent nephrotic proteinuria, we observed 0.05 ± 0.01 PAX2+/SYN+ cells per glomerular section, which corresponded to the regeneration of only 3.3% of lost podocytes (Figures 5G and 5H). Similar results were obtained when the kidney tissues were stained with an anti-WT1 antibody (Figure 5H). Thus, on average, mice with proteinuria remission displayed approximately ten times more PAX2+cell-derived podocytes than mice with persistent proteinuria when all glomeruli of the sections, including those that did not contain PAX2+ cells, were examined (p < 0.05). However, since glomerular damage is focal and segmental, many of the glomeruli did not contain PAX2+cell-derived podocytes or show podocyte depletion. We thus analyzed the percentage of glomeruli presenting PAX2+cell-derived podocytes within the tuft and also the number of PAX2+ cells present within these glomeruli. In the kidney of mice with proteinuria remission, 15.9% ± 1.9% of glomeruli contained on average 2.58 ± 0.18 PAX2+cell-derived podocytes per glomerular section (Figure 5G). By contrast, only 3.6% ± 0.7% of glomeruli contained on average 1.32 ± 0.07 PAX2+cell-derived podocytes per glomerular section in mice with persistent proteinuria (Figure 5G).

Taken together, these results demonstrate that remission or progression of glomerular disorders after podocyte injury depends upon a balance between an efficient and dysfunctional differentiation of PAX2+ progenitors into podocytes.

### GSK3 Inhibition Increases RPC Differentiation into Podocytes through Activation of RARE Transcriptional Activity

**Figure 6:**
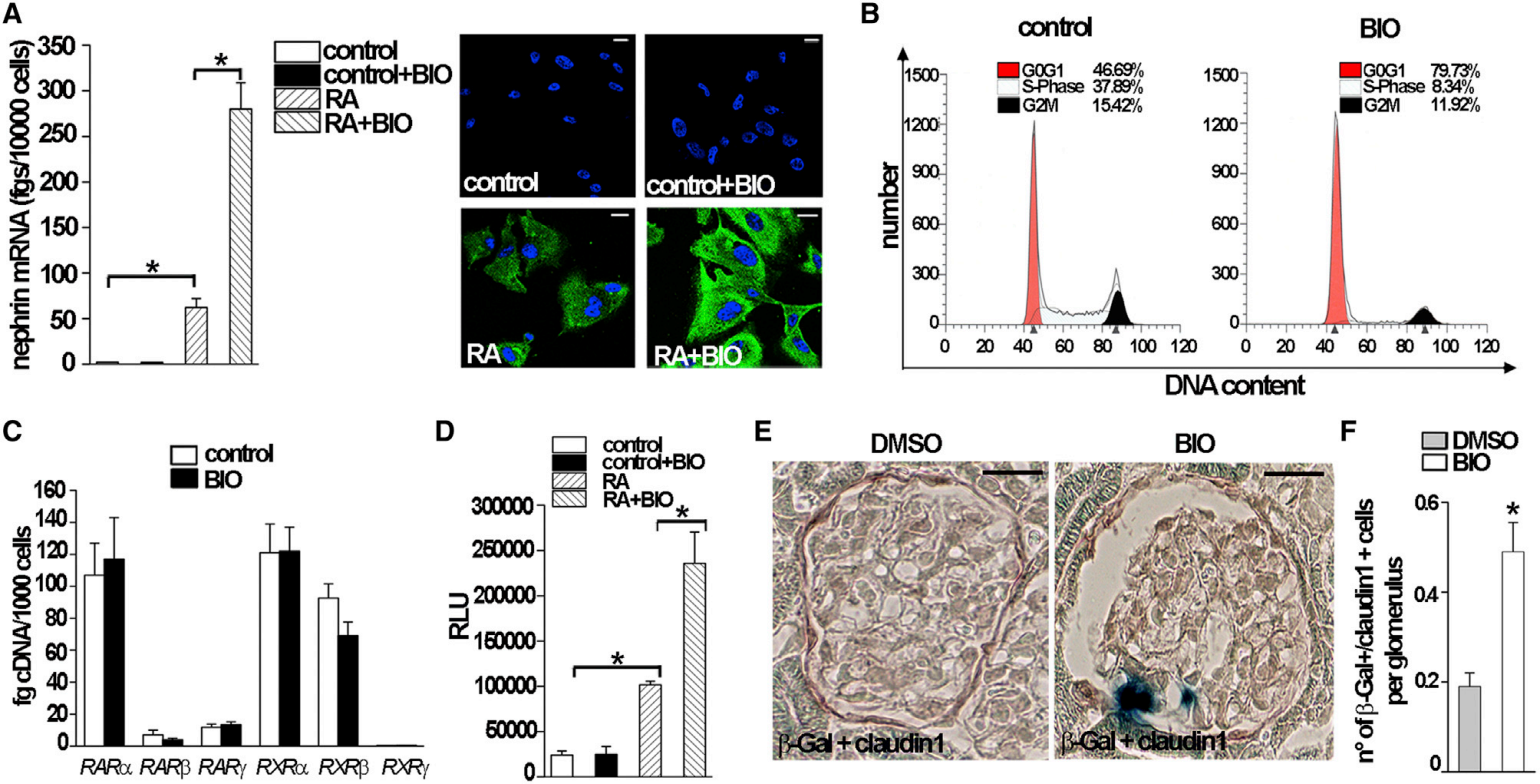
GSK3s Inhibition Increases Podocyte Regeneration from PAX2+ Progenitors by Enhancing RA Transcriptional Activity (A) Effect of BIO treatment on nephrin mRNA (left) and protein (right) expression in hRPCs. Results are expressed as mean ± SEM obtained in at least four separate experiments. ^∗^p < 0.05 by Mann-Whitney test. (B) Representative cell cycle analysis of untreated- (left) and BIO-treated (right) hRPC. One representative experiment out of four independent experiments is shown. (C) Effect of BIO on *RAR/RXR* mRNA expression in hRPC. Results are expressed as mean ± SEM obtained in at least four separate experiments. (D) Effect of BIO treatment on the transcriptional activity of RARE-reporter plasmid in hRPCs. Results are expressed as mean ± SEM obtained in at least four separate experiments ^∗^p < 0.05 by Mann-Whitney test. (E) RARE activity in the glomeruli of *RARE-LacZ* mice with Adriamycin nephropathy treated with DMSO (left) (n = 6) or BIO (right) (n = 6) (β-gal, blue; claudin1, red). (F) Number of β-Gal+/claudin1+ cells lining the Bowman’s capsule or within the glomerular tuft per glomerular section of all glomeruli in DMSO-treated (n = 6) and BIO-treated mice (n = 6). ^∗^p < 0.05 by Mann-Whitney test. Data represent the mean ± SEM. Scale bar, 20 μm.

We then used a described in vitro assay to screen a library of small compounds for their potential to promote human RPC (hRPC) differentiation into podocytes. This assay is based on the analysis of levels of expression of podocyte markers (Ronconi et al., 2009) after exposure to all-trans retinoic acid (RA), which is known to be a powerful inducer of hRPC differentiation into podocytes in culture (Peired et al., 2013). Among the molecules analyzed, we selected the glycogen synthase kinases 3-α and 3-β (GSK3s) inhibitor BIO (6-bromo-indirubin-3′-oxime). Indeed, exposure of hRPCs to 1 μm BIO strongly enhanced the differentiation process in comparison to RA alone, as demonstrated by the higher levels of nephrin mRNA and protein achieved (Figure 6A). We also evaluated the cell-cycle distribution of hRPCs in the presence of BIO. Cell-cycle analysis of untreated and 1 μM BIO-treated cells after 72 hr demonstrated that in BIO-treated hRPCs, the percentage of cells in G0/G1 phase was significantly increased in comparison with untreated cells (45% ± 6% versus 76% ± 8%, p < 0.05; Figure 6B), with a concomitant reduction of the percentage of cells in S phase of the cell cycle (36.5% ± 5.3% versus 13.7% ± 4.2%, p < 0.05; Figure 6B).

Our observation that GSK3 inhibition synergistically enhances RA-induced differentiation suggests that GSK3s may be directly involved in RA receptor activity. qRT-PCR for *RAR* and *RXR* mRNA in BIO-treated and untreated hRPCs did not show any difference in expression of RA receptors (Figure 6C). Because BIO can enhance RA-induced upregulation of *NPHS1* and other podocyte genes acting on RA response elements (RARE) on their promoters, we infected hRPCs with a RARE-reporter luciferase vector. As expected, RA treatment induced activity of the RA-responsive luciferase reporter (Figure 6D), and this effect was markedly enhanced when hRPCs were treated with RA and 1 μM BIO (Figure 6D). Thus, GSK3 inhibition promotes hRPC differentiation toward the podocyte lineage by synchronizing cells in G0/G1 phase of the cell cycle and enhancing RARE transcriptional activity. To investigate the effect of GSK3 inhibition on the enhancement of RA transcriptional activity in vivo, we induced Adriamycin nephropathy in *RARE-lacZ* transgenic mice and treated them with BIO or vehicle (DMSO). As already reported, β-galactosidase (β-gal) activity was not detected within glomeruli of healthy kidneys, where only scattered tubules were stained (Peired et al., 2013). 28 days after induction of podocyte injury, β-gal activity remained scarce in kidneys of DMSO-treated mice, while it appeared in several RPCs identified as claudin1+ cells lining the Bowman’s capsule or within the glomerular tuft of BIO-treated mice (Figures 6E and 6F).

Taken together, these results demonstrate that RPC differentiation into podocytes in vitro can be pharmacologically enhanced by GSK3 inhibition.

### Treatment with GSK3 Inhibitors Increases Podocyte Regeneration and Promotes Glomerular Disease Remission

**Figure 7:**
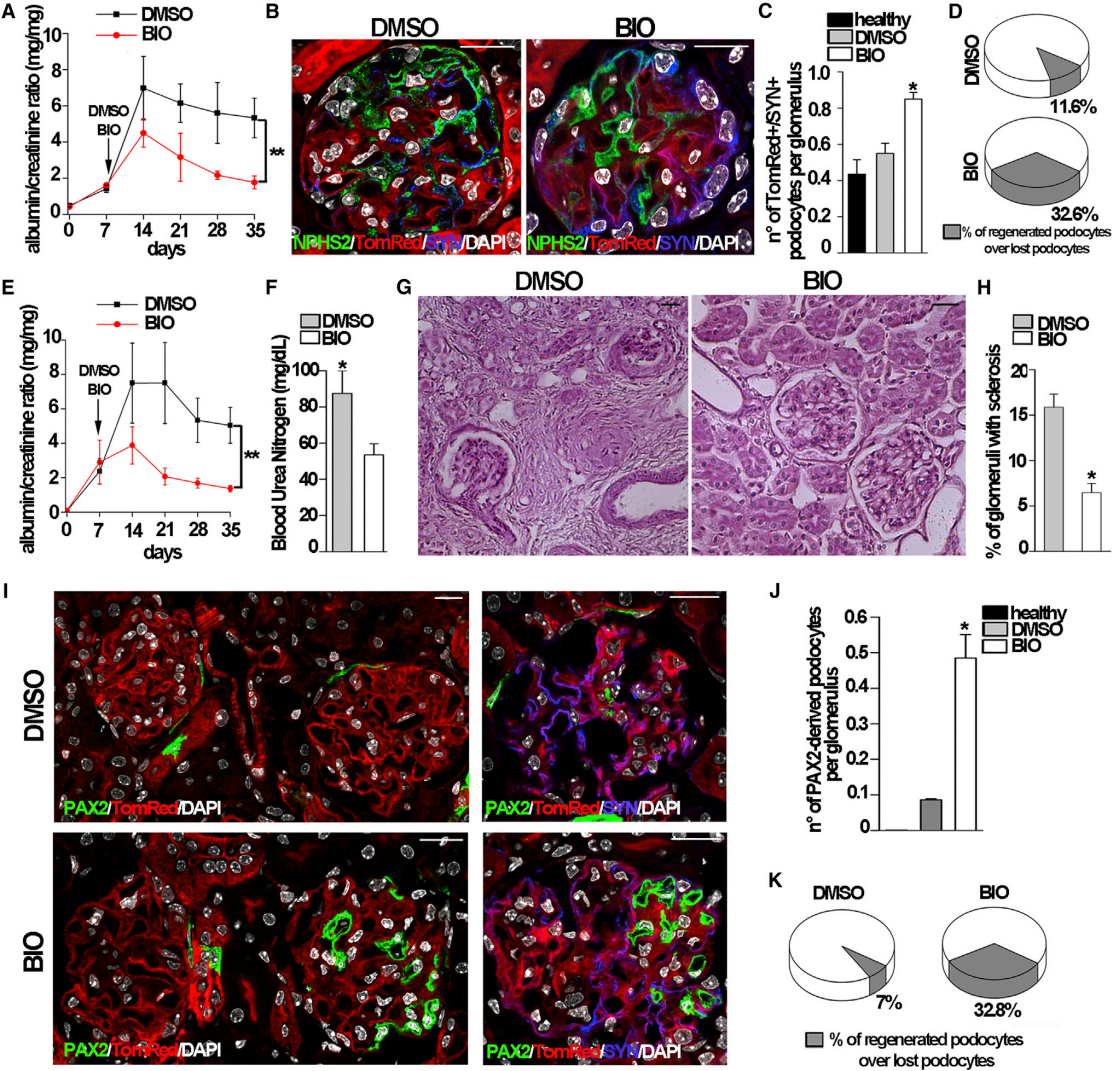
GSK3s Inhibition Induces Remission of Glomerular Disease and Is Associated with the Generation of Novel Podocytes (A) Albumin/creatinine levels in DMSO- (n = 8) and BIO-treated (n = 8) *NPHS2.iCreERT2;mT/mG* mice with Adriamycin nephropathy; day 0 corresponds to the time of second doxorubicin injection. ^∗∗^p < 0.001 by Mann-Whitney test (day 35). (B) Kidney of DMSO- (left) and BIO-treated (right) *NPHS2.iCreERT2;mT/mG* mice stained with anti-SYN (blue). (C) Number of newly generated podocytes per glomerular section of all glomeruli in healthy (n = 4), DMSO-treated (n = 8), and BIO-treated (n = 8) mice. ^∗^p < 0.05 versus healthy and DMSO-treated mice by Mann-Whitney test. (D) Percentage of regenerated podocytes over lost podocytes (n = 8 for DMSO-treated and n = 8 for BIO-treated mice). (E) Albumin/creatinine levels in DMSO- (n = 8) and BIO-treated (n = 8) *Pax2.rtTA;TetO.Cre;mT/mG* mice with Adriamycin nephropathy; day 0 corresponds to the time of second doxorubicin injection. ^∗∗^p < 0.001 (day 35) by Mann-Whitney test. (F) Blood urea nitrogen levels in DMSO- (n = 8), and BIO- (n = 8) treated mice at day 28; ^∗^p < 0.05 versus BIO-treated mice by Mann-Whitney test. (G) Representative periodic acid-Schiff staining of renal sections of DMSO- (left) and BIO-treated (right) mice. (H) Percentage of glomeruli with sclerosis in DMSO- (n = 8) and BIO-treated (n = 8) mice. ^∗^p < 0.05 versus DMSO-treated mice, by Mann-Whitney test. (I) Top: absence of PAX2+ (green) cells in the majority of glomeruli of DMSO-treated *Pax2.rtTA;TetO.Cre;mT/mG* mice (left) and rare PAX2+ (green) cells expressing SYN (blue) inside the glomeruli in DMSO-treated *Pax2.rtTA;TetO.Cre;mT/mG* mice (right). Bottom: a higher number of PAX2+ cells inside the glomeruli in BIO-treated *Pax2.rtTA;TetO.Cre;mT/mG* mouse (left) and PAX2+ cells inside the glomeruli express SYN (blue) (right). (J) Quantification of PAX2+/SYN+ cells per glomerular section of all glomeruli in healthy (n = 4), DMSO-treated (n = 8), and BIO-treated (n = 8) mice. ^∗^p < 0.05 versus healthy and DMSO-treated mice. (K) Percentage of PAX2-derived podocytes over lost podocytes (n = 8 for DMSO- and n = 8 for BIO-treated mice). Data represent the mean ± SEM. DAPI counterstains nuclei. Scale bar, 20 μm.

To evaluate whether the effects of BIO on RPC differentiation result in an improvement of renal regeneration following acute podocyte loss in vivo, we induced Adriamycin nephropathy in *NPHS2.iCreER*^*T2*^*;mT/mG* mice, and when a significant peak in proteinuria was observed (day 7 starting from the second doxorubicin injection), we divided the mice into groups exhibiting comparable proteinuria and started DMSO or BIO treatment. The urinary albumin/creatinine ratio was monitored for the following 28 days, then animals were sacrificed and kidneys harvested for confocal microscopy analysis. The number of BIO-treated animals with proteinuria decreased after 14 days of BIO treatment and remained significantly lower than that in the control group at all following time points (Figure 7A). Representative images of glomeruli of DMSO- and BIO-treated *NPHS2.iCreER*^*T2*^*;mT/mG* mice are shown in Figure 7B. We counted 9.1 ± 0.12 GFP+/SYN+ podocytes per glomerular section in DMSO-treated mice and 9.5 ± 0.14 GFP+/SYN+ podocytes per glomerular section in BIO-treated animals (not significant). By contrast, the number of newly generated podocytes was statistically different in the two groups of mice. Indeed, we counted 0.55 ± 0.05 TomRed+/SYN+ podocytes per section in DMSO-treated mice and 0.85 ± 0.03 TomRed+/SYN+ podocytes per glomerular section in BIO-treated mice (Figure 7C). These results demonstrate that in DMSO-treated mice, only 11.6% of the ablated cells had been replaced, while this percentage increased to 32.6% in BIO-treated mice (Figure 7D). Similar results were obtained when the kidney tissues were stained with an anti-WT1 antibody (13.2% in DMSO-treated mice versus 37.3% in BIO-treated mice). Significantly, all eight mice treated with BIO underwent proteinuria remission, in comparison to only two of the DMSO-treated mice (p = 0.007, χ^2^ test with Fisher’s correction). To evaluate if the increased podocyte regeneration induced by BIO treatment was related to an increased differentiation of RPCs into podocytes, the same experiments were repeated in *Pax2.rtTA;TetO.Cre;mT/mG* mice. The albumin/creatinine ratio confirmed that all BIO-treated mice underwent proteinuria remission (Figure 7E) associated with a statistically significant decrease in BUN levels at day 28 in comparison to DMSO-treated mice (Figure 7F). Treatment with BIO reduced glomerulosclerosis in comparison to DMSO treatment (Figure 7G), as evidenced by the reduction in the percentage of glomeruli with sclerosis (Figure 7H). Confocal microscopy clearly demonstrated the presence of a significantly higher number of newly generated podocytes (PAX2+/SYN+ cells) within glomeruli of BIO-treated mice in comparison to DMSO-treated animals (Figure 7I). In DMSO-treated mice, the number of green PAX2+/SYN+ cells in the tuft was 0.086 ± 0.01 cells/glomerular section (Figure 7J), which corresponded to the regeneration of 7% of lost podocytes (Figure 7K). By contrast, in BIO-treated mice, the number of green PAX2+/SYN+ cells increased to 0.48 ± 0.06 cells per glomerular section (Figure 7J), which corresponded to an average regeneration of 32.8% of lost podocytes (Figure 7K). Similar results were obtained when the kidney tissues were stained with an anti-WT1 antibody (10.3% in DMSO-treated mice versus 35.8% in BIO-treated mice). Significantly, all eight mice treated with BIO underwent proteinuria remission, in comparison to only one of the DMSO-treated mice (p = 0.001, χ^2^ test with Fisher’s correction). Taken together, these results demonstrate that differentiation of RPCs into podocytes can be pharmacologically enhanced through treatment with GSK3 inhibitors.

## Discussion

Determinants of outcome in patients with CKD remain mostly unknown. Here, we demonstrate that an efficient generation of novel podocytes by RPCs critically influences the course of CKD and that RPC differentiation into podocytes can be pharmacologically enhanced for therapeutic purposes.

Until now, the role of RPCs in glomerular disease was uncertain, mostly because of the lack of a specific marker to specifically identify and trace the murine homolog of the human population (Shankland et al., 2014; Romagnani et al., 2015). The results of this study show that PAX2, which is specifically co-expressed with CD133 in human RPCs (Romagnani et al., 2015), traces the mouse counterpart of human RPCs within the Bowman’s capsule and that ∼8%–10% of total podocytes observed in the adult mouse kidney are derived from PAX2+ PECs after birth. These results demonstrate that PAX2+ PECs behave as podocyte progenitors, even once glomerular development has ended, and contribute to the increase of kidney dimension that is observed during childhood and adolescence. In adult animals, PAX2+ cells remained mostly localized within the Bowman’s capsule of subcapsular glomeruli, but in only ∼30% of juxtamedullary glomeruli. This is also in agreement with the observation that the regenerative capacity of these glomeruli is more limited (Ihara et al., 2010) and that sclerosis initiates and is more frequent in juxtamedullary glomeruli (Ikoma et al., 1990).

By using this RPC-specific tracking tool, we could also establish that RPC differentiation into podocytes can occur following glomerular injury and critically contributes to disease improvement. Previous studies concluded that no podocyte turnover occurs during aging and following nephrectomy (Berger et al., 2014), but the role of RPCs was never explored in models of podocyte injury. Generation of new podocytes was reported by Wanner et al. once podocyte depletion was induced through diphtheria toxin injection (Wanner et al., 2014), but the authors could not establish from where these new podocytes were derived or whether they could influence the course of the disease. To provide answers to these questions, in this study, we induced podocyte depletion by doxorubicin treatment. Adriamycin nephropathy mimics human FSGS and can exhibit a variable outcome over time, which is consistent with what happens in patients. Indeed, even if they are affected by the same disorder, some mice develop a persistent proteinuria and CKD, while others show a peak of proteinuria that is followed by proteinuria remission, maintenance of kidney function, and disease resolution (Peired et al., 2013).

To establish whether replacement of lost podocytes by RPCs could influence CKD outcome, we traced podocyte regeneration to compare mice that underwent proteinuria remission with those that developed persistent proteinuria and CKD using two different lineage-tracing strategies. By tracking podocytes or RPCs, we consistently observed that proteinuria remission was associated with regeneration by RPCs of 5%–10% of total kidney podocytes. Since nephrotic syndrome in these mice caused a 15%–20% podocyte loss, this corresponds to regeneration of approximately one-third of lost podocytes. By contrast, in mice that developed persistent proteinuria and CKD, podocyte regeneration was irrelevant. The *Pax2.rtTA;TetO.Cre;mT/mG* model also allowed us to establish that RPCs are the source of podocyte regeneration after injury. Indeed, in mice that underwent proteinuria remission after injury, ∼30% of lost podocytes were replaced by new podocytes generated by PAX2+ cells. By contrast, the percentage of PAX2+cell-derived podocytes was irrelevant in mice with persistent proteinuria. The virtually identical results observed once the same experimental strategy was established in *Pax2.rtTA;TetO.Cre;mT/mG* as well as in *NPHS2.iCreER*^*T2*^*;mT/mG* mice demonstrates that RPCs are the source of podocyte regeneration.

From pathology studies in transplanted patients, we know that an increase of 20% of podocyte mass can efficiently compensate a reduction of 50% of filtration surface (Song et al., 2014). Thus, the amount of podocyte replacement that can be provided by RPCs represents a substantial possibility of recovery that can critically determine the outcome of a glomerular disorder. Consistently, we know that the glomerulus can recover from up to a 20% reduction in podocyte density occurring over a short period of time (Wiggins, 2007). By contrast, as >30% podocyte depletion occurs, glomerular stress and further podocyte depletion supervene, triggering glomerulosclerosis and ESRD (Fukuda et al., 2012).

Taken together, these results demonstrate that when RPCs acquire phenotypic and functional features of fully differentiated podocytes, proteinuria remission occurs, while when progenitor differentiation is not effective, proteinuria persists and glomerular scars emerge. The pivotal role played by PECs in the pathogenesis of intraglomerular scars in FSGS was recently suggested by several studies (Shankland et al., 2014; Smeets et al., 2009; Lasagni and Romagnani, 2010). The results of this study demonstrate that glomerular scars are caused by an inefficient differentiation into mature podocytes of those PECs that represent RPCs. Based on this observation, we reasoned that RPCs may represent a therapeutic target and that enhancing RPC differentiation into podocytes could reduce formation of glomerular scars and promote podocyte regeneration and proteinuria remission. To evaluate if enhancement of RPC differentiation into podocytes may represent an attractive therapeutic strategy to promote remission of glomerular disorders, we screened a library of small molecules for their potential to promote RPC differentiation into podocytes. Among the molecules analyzed, we identified the GSK3 inhibitor BIO as a strong promoter of hRPC differentiation toward podocytes in vitro and in vivo. We also demonstrated that BIO acts by increasing RA binding to its specific RARE elements and by enhancing RPC sensitivity to the differentiation effect of endogenously produced RA. Indeed, RA is a podocyte differentiation factor that is released within the Bowman’s space following glomerular injury (Peired et al., 2013). Interestingly, it was previously shown that albuminuria sequesters RA within the Bowman’s space and administration of RA reduces proteinuria in mice with Adriamycin nephropathy (Peired et al., 2013). However, doses of RA that are toxic in humans are needed to rescue the effects of albuminuria (Tallman et al., 2000). Enhancement of RPC differentiation into podocytes by using BIO avoided toxicity and significantly improved the disease outcome. This observation demonstrates that the course of CKD can be shifted from progression to remission by acting on the RPC response to injury.

Several previous studies support the possibility that RPC differentiation into podocytes may be involved in remission of different types of diseases, including proliferative glomerulonephritis (Rizzo et al., 2013), gestational pre-eclampsia (Penning et al., 2014), and diabetic nephropathy (Pichaiwong et al., 2013). In addition, drugs that are already used in clinical practice to delay disease progression, such as renin-angiotensin-aldosterone system blockers, not only prevent progressive renal damage but also promote the regression of glomerulosclerosis in several models of CKD (Remuzzi et al., 2006), suggesting that they may also exert their beneficial effects by promoting RPC differentiation into podocytes (Benigni et al., 2011). In addition, leptin replacement promotes disease regression in animal models of advanced diabetic nephropathy by increasing podocyte number, another effect that may reasonably be mediated by podocyte regeneration provided by RPCs (Pichaiwong et al., 2013). Further studies are necessary to verify these points. However, the observation that an efficient differentiation of RPCs into podocytes determines the outcome of glomerular disorders and that this process can be pharmacologically enhanced has important implications for the treatment of patients with CKD.

## Experimental Procedures

### NPHS2.iCreER^T2^;mT/mG Mice

*NPHS2.iCreER*^*T2*^ mice were a kind gift from Dr. Farhad Danesh (Bayor College of Medicine, Houston TX, USA), and *B6.129(Cg)-Gt(ROSA)26Sor*^*tm4(ACTB-tdTomato,-EGFP)Luo*^*/J* (here abbreviated mT/mG) mice were purchased from Jackson Laboratory. Double-transgenic mice were obtained by crossing the two lines on a 100% C57Bl/6 background. For information on genotyping, induction procedures, and tissue processing, see Supplemental Experimental Procedures.

### Pax2.rtTA;TetO.Cre;mT/mG Mice and Pax2.rtTA;TetO.Cre;R26.Confetti Mice

Pax2.rtTA;TetO.Cre;mT/mG mice or Pax2.rtTA;TetO.Cre;R26.Confetti mice were developed by crossing the mT/mG reporter strain B6.129(Cg)-Gt(ROSA)26Sor^tm4(ACTB-tdTomato,-EGFP)Luo^/J or the Confetti strain Gt(ROSA)26Sor^tm1(CAG-Brainbow2.1)Cle^/J with the TetO.Cre strain B6.Cg-Tg(TetO-Cre)1Jaw/J, both purchased from Jackson Laboratory. Double-transgenic mice were then crossed with Pax2.rtTA mice (Burger et al., 2011) to obtain a triple-transgenic inducible mouse model with a 100% C57Bl/6 background (Figures S1A and S2A). For information on genotyping, induction procedures, and tissue processing, see Supplemental Experimental Procedures.

### Adriamycin Nephropathy and BIO Treatment

Adriamycin nephropathy and BIO treatment are detailed in Supplemental Experimental Procedures.

### Immunofluorescence and Confocal Microscopy

Confocal microscopy was performed on 10-μm sections of renal frozen tissues or on cells by using a Leica SP5 AOBS confocal microscope (Leica) equipped with a Chameleon Ultra-II two-photon laser (Coherent). Detailed procedures are reported in Supplemental Experimental Procedures.

### Podocyte Quantification

For quantitation of podocytes in *NPHS2.iCreERT2;mT/mG* mice, the number of podocytes (green cells positively stained with SYN or WT1) and newly generated podocytes (red cells positively stained with SYN or WT1) per glomerular section was evaluated in at least 50 glomeruli of at least four sections for each mouse by two independent observers. Similarly, in *Pax2.rtTA;TetO.Cre;mT/mG* mice, the number of new podocytes generated by PAX2+ renal progenitors was evaluated by counting the number of green cells positively stained with SYN or WT1 per glomerular section in at least 50 glomeruli of at least four sections for each mouse by two independent observers. For detailed calculations, see Supplemental Experimental Procedures.

### hRPC Cultures and In Vitro Differentiation

For podocyte differentiation and evaluation of BIO effects, cells were treated for 48 hr with DMEM-F12 (Sigma-Aldrich) supplemented with 10% fetal bovine serum (FBS) (Hyclone, from Euroclone) and 100 μM RA (Sigma-Aldrich) in the presence or absence of 1 μM BIO. The expression of nephrin at the mRNA level was evaluated by real-time qRT-PCR, and the expression of nephrin at protein levels was evaluated by immunofluorescence. Procedures for small-compound library screening, cell-cycle analysis, real-time qRT-PCR, and hRPC infection are detailed in Supplemental Experimental Procedures.

### Study Approval

Animal experiments were approved by the institutional review board and performed in accordance with institutional, regional, and state guidelines and in adherence to the NIH’s *Guide for the Care and Use of Laboratory Animals*. Mice were housed in a specific-pathogen-free facility with free access to chow and water and a 12 hr day/night cycle. hRPCs were obtained as previously described (Sagrinati et al., 2006; Ronconi et al., 2009) in agreement with the ethics committee on human experimentation of the Azienda Ospedaliero-Universitaria Careggi, Florence, Italy. Informed consent was obtained from each patient involved in the study.

### Statistics

The results are expressed as mean ± SEM. Comparison between groups was performed by the Mann-Whitney test, the Wilcoxon test, or ANOVA for multiple comparisons (repeated measures) with Bonferroni post hoc analysis. Frequencies were compared by χ^2^ test with Fisher’s correction when appropriate. p < 0.05 was considered to be statistically significant.

## Author Contributions

L.L. and P.R. conceived of the study. M.L.A. and F.B. designed and performed immunofluorescence and confocal microscopy. E.R., D.L., S.N., S.R. and A.P. designed and performed experiments in the mouse systems. B.M. and A.S. designed and performed in vitro experiments. A. Burger and B.S. provided the PAX2.rtTA mice. A. Buccoliero performed histologic staining. L.L., M.L.A., E.L. and P.R. analyzed the data. L.L. and P.R. wrote the paper with input from all authors.

## Supplemental Information

Document S1. Supplemental Experimental Procedures and Figures S1 and S2

Movie S1. PAX2+ Progenitors of the Bowman’s Capsule Generate New Podocytes, Related to Figure 4

Document S2. Article plus Supplemental Information

## References

[bib1] Appel D., Kershaw D.B., Smeets B., Yuan G., Fuss A., Frye B., Elger M., Kriz W., Floege J., Moeller M.J. (2009). Recruitment of podocytes from glomerular parietal epithelial cells. J. Am. Soc. Nephrol..

[bib2] Benigni A., Morigi M., Rizzo P., Gagliardini E., Rota C., Abbate M., Ghezzi S., Remuzzi A., Remuzzi G. (2011). Inhibiting angiotensin-converting enzyme promotes renal repair by limiting progenitor cell proliferation and restoring the glomerular architecture. Am. J. Pathol..

[bib3] Berger K., Schulte K., Boor P., Kuppe C., van Kuppevelt T.H., Floege J., Smeets B., Moeller M.J. (2014). The regenerative potential of parietal epithelial cells in adult mice. J. Am. Soc. Nephrol..

[bib4] Burger A., Koesters R., Schäfer B.W., Niggli F.K. (2011). Generation of a novel rtTA transgenic mouse to induce time-controlled, tissue-specific alterations in Pax2-expressing cells. Genesis.

[bib5] Bussolati B., Bruno S., Grange C., Buttiglieri S., Deregibus M.C., Cantino D., Camussi G. (2005). Isolation of renal progenitor cells from adult human kidney. Am. J. Pathol..

[bib6] Chen Y.M., Miner J.H. (2012). Glomerular basement membrane and related glomerular disease. Transl. Res..

[bib7] Dressler G.R. (2008). Epigenetics, development, and the kidney. J. Am. Soc. Nephrol..

[bib8] Eckardt K.U., Coresh J., Devuyst O., Johnson R.J., Köttgen A., Levey A.S., Levin A. (2013). Evolving importance of kidney disease: from subspecialty to global health burden. Lancet.

[bib9] Fukuda A., Chowdhury M.A., Venkatareddy M.P., Wang S.Q., Nishizono R., Suzuki T., Wickman L.T., Wiggins J.E., Muchayi T., Fingar D. (2012). Growth-dependent podocyte failure causes glomerulosclerosis. J. Am. Soc. Nephrol..

[bib10] Hartman H.A., Lai H.L., Patterson L.T. (2007). Cessation of renal morphogenesis in mice. Dev. Biol..

[bib11] Ihara G., Kiyomoto H., Kobori H., Nagai Y., Ohashi N., Hitomi H., Nakano D., Pelisch N., Hara T., Mori T. (2010). Regression of superficial glomerular podocyte injury in type 2 diabetic rats with overt albuminuria: effect of angiotensin II blockade. J. Hypertens..

[bib12] Ikoma M., Yoshioka T., Ichikawa I., Fogo A. (1990). Mechanism of the unique susceptibility of deep cortical glomeruli of maturing kidneys to severe focal glomerular sclerosis. Pediatr. Res..

[bib13] Kazimierczak J. (1980). A study of scanning (SEM) and transmission (TEM) electron microscopy of the glomerular capillaries in developing rat kidney. Cell Tissue Res..

[bib14] KDIGO (Kidney Disease: Improving Global Outcomes) Glomerulonephritis Work Group (2012). KDIGO clinical practice guideline for glomerulonephritis. Kidney Int. Suppl..

[bib15] Kriz W., LeHir M. (2005). Pathways to nephron loss starting from glomerular diseases-insights from animal models. Kidney Int..

[bib16] Lasagni L., Romagnani P. (2010). Glomerular epithelial stem cells: the good, the bad, and the ugly. J. Am. Soc. Nephrol..

[bib17] Lasagni L., Lazzeri E., Shankland S.J., Anders H.J., Romagnani P. (2013). Podocyte mitosis - a catastrophe. Curr. Mol. Med..

[bib18] Lee V.W., Harris D.C. (2011). Adriamycin nephropathy: a model of focal segmental glomerulosclerosis. Nephrology (Carlton).

[bib19] Peired A., Angelotti M.L., Ronconi E., la Marca G., Mazzinghi B., Sisti A., Lombardi D., Giocaliere E., Della Bona M., Villanelli F. (2013). Proteinuria impairs podocyte regeneration by sequestering retinoic acid. J. Am. Soc. Nephrol..

[bib20] Penning M.E., Bloemenkamp K.W., van der Zon T., Zandbergen M., Schutte J.M., Bruijn J.A., Bajema I.M., Baelde H.J. (2014). Association of preeclampsia with podocyte turnover. Clin. J. Am. Soc. Nephrol..

[bib21] Pichaiwong W., Hudkins K.L., Wietecha T., Nguyen T.Q., Tachaudomdach C., Li W., Askari B., Kobayashi T., O’Brien K.D., Pippin J.W. (2013). Reversibility of structural and functional damage in a model of advanced diabetic nephropathy. J. Am. Soc. Nephrol..

[bib22] Remuzzi G., Benigni A., Remuzzi A. (2006). Mechanisms of progression and regression of renal lesions of chronic nephropathies and diabetes. J. Clin. Invest..

[bib23] Rizzo P., Perico N., Gagliardini E., Novelli R., Alison M.R., Remuzzi G., Benigni A. (2013). Nature and mediators of parietal epithelial cell activation in glomerulonephritides of human and rat. Am. J. Pathol..

[bib24] Romagnani P. (2009). Toward the identification of a “renopoietic system”?. Stem Cells.

[bib25] Romagnani P., Lasagni L., Remuzzi G. (2013). Renal progenitors: an evolutionary conserved strategy for kidney regeneration. Nat. Rev. Nephrol..

[bib26] Romagnani P., Rinkevich Y., Dekel B. (2015). The use of lineage tracing to study kidney injury and regeneration. Nat. Rev. Nephrol..

[bib27] Ronconi E., Sagrinati C., Angelotti M.L., Lazzeri E., Mazzinghi B., Ballerini L., Parente E., Becherucci F., Gacci M., Carini M. (2009). Regeneration of glomerular podocytes by human renal progenitors. J. Am. Soc. Nephrol..

[bib28] Ryan G., Steele-Perkins V., Morris J.F., Rauscher F.J., Dressler G.R. (1995). Repression of Pax-2 by WT1 during normal kidney development. Development.

[bib29] Sagrinati C., Netti G.S., Mazzinghi B., Lazzeri E., Liotta F., Frosali F., Ronconi E., Meini C., Gacci M., Squecco R. (2006). Isolation and characterization of multipotent progenitor cells from the Bowman’s capsule of adult human kidneys. J. Am. Soc. Nephrol..

[bib30] Schieppati A., Remuzzi G. (2005). Chronic renal diseases as a public health problem: epidemiology, social, and economic implications. Kidney Int. Suppl..

[bib31] Shankland S.J., Smeets B., Pippin J.W., Moeller M.J. (2014). The emergence of the glomerular parietal epithelial cell. Nat. Rev. Nephrol..

[bib32] Short K.M., Combes A.N., Lefevre J., Ju A.L., Georgas K.M., Lamberton T., Cairncross O., Rumballe B.A., McMahon A.P., Hamilton N.A. (2014). Global quantification of tissue dynamics in the developing mouse kidney. Dev. Cell.

[bib33] Smeets B., Angelotti M.L., Rizzo P., Dijkman H., Lazzeri E., Mooren F., Ballerini L., Parente E., Sagrinati C., Mazzinghi B. (2009). Renal progenitor cells contribute to hyperplastic lesions of podocytopathies and crescentic glomerulonephritis. J. Am. Soc. Nephrol..

[bib34] Song T., Fu L., Huang Z., He S., Zhao R., Lin T., Wei Q. (2014). Change in renal parenchymal volume in living kidney transplant donors. Int. Urol. Nephrol..

[bib35] Tallman M.S., Andersen J.W., Schiffer C.A., Appelbaum F.R., Feusner J.H., Ogden A., Shepherd L., Rowe J.M., François C., Larson R.S., Wiernik P.H. (2000). Clinical description of 44 patients with acute promyelocytic leukemia who developed the retinoic acid syndrome. Blood.

[bib36] Wanner N., Hartleben B., Herbach N., Goedel M., Stickel N., Zeiser R., Walz G., Moeller M.J., Grahammer F., Huber T.B. (2014). Unraveling the role of podocyte turnover in glomerular aging and injury. J. Am. Soc. Nephrol..

[bib37] Wiggins R.C. (2007). The spectrum of podocytopathies: a unifying view of glomerular diseases. Kidney Int..

[bib38] Ye Y., Wang B., Jiang X., Hu W., Feng J., Li H., Jin M., Ying Y., Wang W., Mao X., Jin K. (2011). Proliferative capacity of stem/progenitor-like cells in the kidney may associate with the outcome of patients with acute tubular necrosis. Hum. Pathol..

