# Supplementary material for: Podocyte Regeneration Driven by Renal Progenitors Determines Glomerular Disease Remission and Can Be Pharmacologically Enhanced
Source: Stem Cell Reports. 2015 Jul 30;5(2):248–63. doi: 10.1016/j.stemcr.2015.07.003 (PMC4618832; doi:10.1016/j.stemcr.2015.07.003)
Supplement: Document S1. Supplemental Experimental Procedures and Figures S1 and S2 [file mmc1.pdf]

Stem Cell Reports, Volume *5*

Supplemental Information

Podocyte Regeneration Driven by Renal Progenitors Determines Glomerular Disease Remission and Can Be Pharmacologically Enhanced

Laura Lasagni, Maria Lucia Angelotti, Elisa Ronconi, Duccio Lombardi, Sara Nardi, Anna Peired, Francesca Becherucci, Benedetta Mazzinghi, Alessandro Sisti, Simone Romoli, Alexa Burger, Beat Schaefer, Annamaria Buccoliero, Elena Lazzeri, and Paola Romagnani

Supplemental Figures

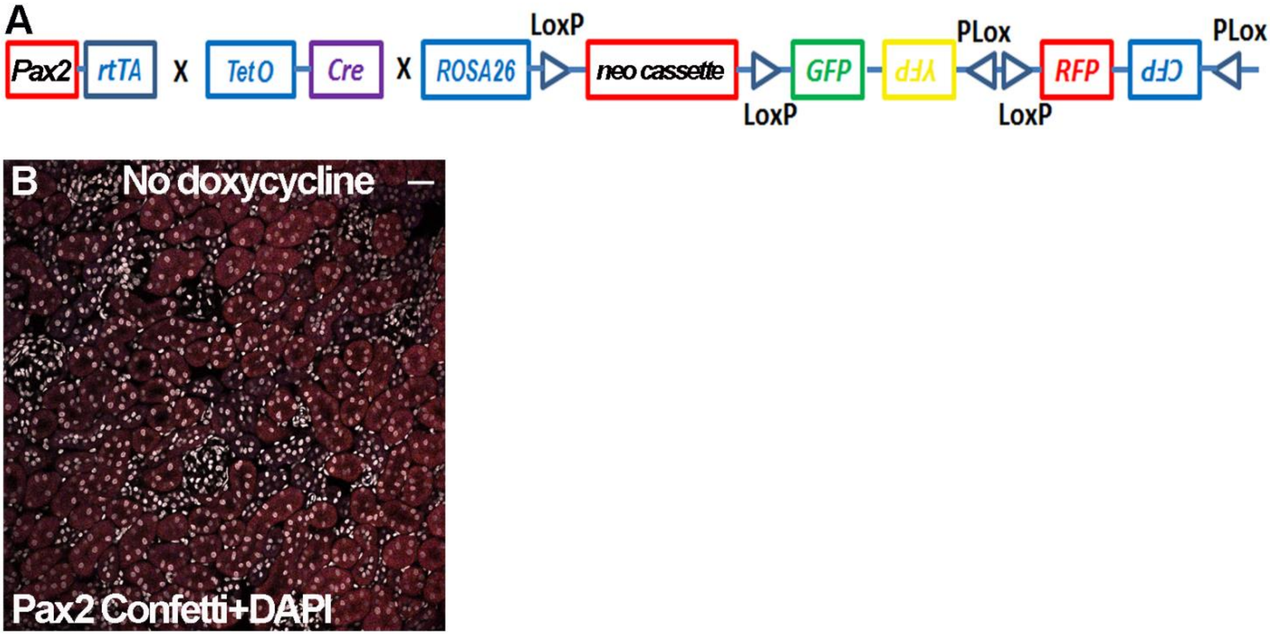

Figure S1 The inducible *Pax2.rtTA;TetO.Cre;R26.Confetti*, related to Figure 3. (A) The inducible *Pax2.rtTA;TetO.Cre;R26.Confetti* mouse is produced by crossing the *Pax2-rtTA* transgenic mice with *TetOnCre* and *Rosa26-Confetti* transgenic mice. The triple transgenic mouse constitutively expresses rtTA in PAX2+ cells but does not express the reporter proteins in any cell type while maintained on water not containing doxycyclin. When doxyclin is included in the water, PAX2+ cells express rtTA that binds the TetO element so that Cre recombinase expression is induced. The Cre protein will specifically cut out or invert the floxed fluorochrome sequences and then turn on GFP, YFP, CFP or RFP expression. Even after withdrawal of doxycyclin from the water, these PAX2+ cells will permanently express the reporters, whereas any new PAX2+ cells that develop after dox exposure will not express the fluorescent reporters. (B) Absence of basal Cre recombinase activity in the kidney of *Pax2.rtTA;TetO.Cre;R26.Confetti* healthy mice (n=3) analysed at 5 weeks of age. Scale bar 20 µm.

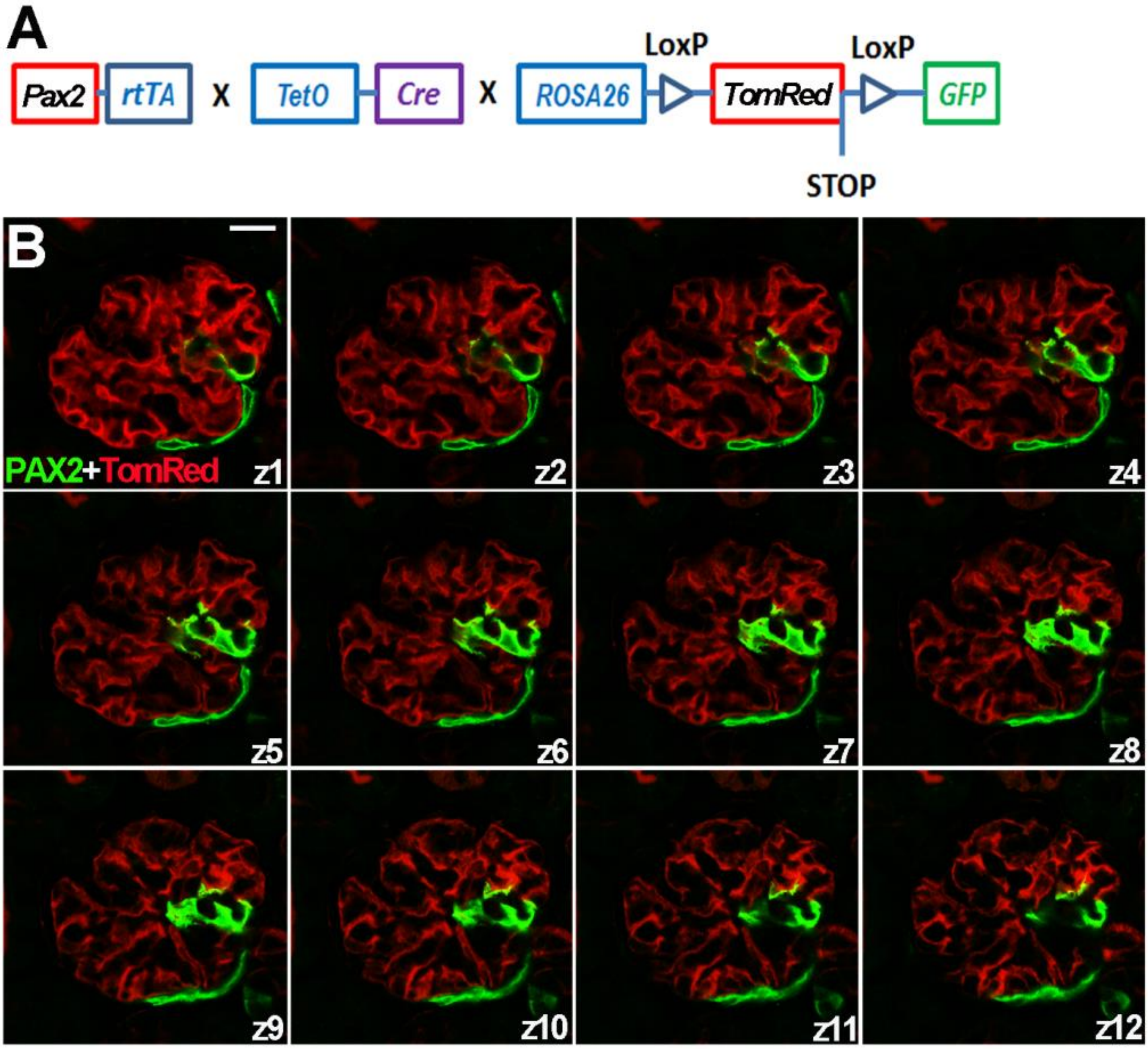

Figure S2 PAX2+ progenitors of Bowman's capsule generate new podocytes, related to Figure 4. (A) The inducible *Pax2.rtTA;TetO.Cre;mT/mG* mice were developed by crossing the *Pax2-rtTA* transgenic mice with the *TetOCre* and *Rosa26-mT/mG* reporter strain. The triple transgenic mouse constitutively expresses rtTA in PAX2+ cells, and the TomRed protein in any cell type, but does not express the reporter protein GFP while maintained on water without doxycyclin. When doxycyclin is included in the water, PAX2+ cells express rtTA that binds the TetO element so that Cre expression is induced. The Cre protein will specifically cut out the floxed TomRed sequence turning on GFP expression. Even after withdrawal of doxycyclin from the water, these PAX2+ cells will permanently express the reporters, whereas any new PAX2+ cells that develop after doxycyclin exposure will express the TomRed but not the GFP reporter. (B) Z-section stack of a glomerulus of a *Pax2.rtTA;TetO.Cre;mT/mG* mouse undergoing remission of proteinuria showing the

continuity of an intraglomerular PAX2-derived cell with the PEC of Bowman's capsule. Images were taken from a 30 µm kidney slice, 1 µm optical slice thickness, 12 z-sections collected at 1 µm intervals. Scale bar 20 µm.

Movie S1 PAX2+ progenitors of Bowman's capsule generate new podocytes, related to Figure 4. Forty-one z-sections collected at 0.64 µm intervals from the same glomerulus of a *Pax2.rtTA;TetO.Cre;mT/mG* mouse undergoing remission of proteinuria are showed.

Supplemental Experimental Procedures

***NPHS2.iCreER^{T2};mT/mG* mice.** The *NPHS2.iCreERT2;mT/mG* mice were developed to stably and irreversibly tag podocytes upon induction with tamoxifen. These mice express strong red fluorescence (Tomato Red, mT) in all tissues and cell types, but the *NPHS2.iCre* promoter, following tamoxifen administration, drives the expression of the Cre recombinase only in podocytes, allowing the expression of the membrane-targeted GFP (mG) and marking only these cells in green. Following tamoxifen withdrawal, new podocytes will be labelled with mT. Reporter transgene expression was induced by i.p. injection with 2 mg/mouse tamoxifen (Sigma-Aldrich, St. Louis, MO, USA) for 8 days. At sacrifice, kidneys were collected following cardiac perfusion with 4% paraformaldehyde (PFA, Sigma-Aldrich) in PBS then immersed in a 15% sucrose solution in PBS for 2 h at 4°C and subsequently in a 30% sucrose solution in PBS overnight at 4°C, then frozen and a small portion was included in paraffin for microscopic analysis.

***Pax2.rtTA;TetO.Cre;mT/mG* mice and *Pax2.rtTA;TetO.Cre;R26.Confetti* mice.** Reporter transgene recombination was induced at 5 weeks of age by administration of doxycycline hyclate (2 mg/ml, Sigma-Aldrich) in drinking water additioned of 2.5% sucrose (Sigma-Aldrich), for 10 days. Mice were sacrificed at the end of the induction period (n=4) or following a washout period of 9 month (n=6), kidneys were collected, incubated in 4% PFA in PBS for 2 h at 4°C followed by immersion in a 15% sucrose solution in PBS for 2 h at 4°C and subsequently in a 30% sucrose solution in PBS overnight at 4°C, then frozen.

To track PAX2 expressing cells in postnatal kidneys, *Pax2.rtTA;TetO.Cre;R26.Confetti* mice were induced at P5 by administering doxycycline in drinking water to the mothers for 10 days. Pups were sacrificed at day 14 (n=8) or at 5 weeks of age (n=8), kidneys collected as reported and analyzed by confocal microscopy.

Basal Cre recombinase activity (leakage) in absence of doxycycline induction, was assessed in healthy *Pax2.rtTA;TetO.Cre;mT/mG* mice (n=3) at 12 weeks of age (Figure 4G), in healthy *Pax2.rtTA;TetO.Cre;R26.Confetti* mice (n=3) at 5 weeks of age (Figure S1B) and in doxorubicin-treated *Pax2.rtTA;TetO.Cre;mT/mG* mice (n=3) (Figure 4H). To this aim, Adriamycin nephropathy was induced as described below in mice that did not receive doxycycline; animals were sacrificed at day 28 following the second doxorubicin injection, kidneys were collected as detailed above and analyzed by confocal microscopy.

Genotyping Tail biopsies were incubated overnight at 55°C in lysis reagent (1M TrisHCl pH 8.5; 0.5 M EDTA, 20% SDS, 4M NaCl, 0.1 mg/ml proteinase K neutralized with 40 mM TrisHCl), centrifuged and DNA extracted using isopropanol (Sigma-Aldrich). DNA was resuspended in PCR grade water and used for PCR with the following primers and parameters: *Pax2.rtTA* forward 5'-AACGCACTGTACGCTCTGTC-3' and reverse 5'-GAATCGGTGGTAGGTGTCTC-3', 5 min 94°C, 35 cycles of 30 sec 94°C, 30 sec 53°C, 30 sec 72°C and, finally, 7 min 72°C; *NPHS2.iCRE*^{TR2} forward 5'-TCAACATGCTGCACAGGAGAT-3' and reverse 5'-ACCATAGATCAGGCGGTGGGT-3'; 4 min 94°C, 30 cycles of 94°C 30 sec, 57°C 30 sec, 72°C and, finally, 72°C for 10 minutes. Primers and PCR parameters for the *TetO.Cre*, *mT/mG* and Confetti strains were obtained from Jackson Laboratory online resources protocols of the relative strain purchased.

***RARE-lacZ* transgenic mice** *RARE-lacZ* transgenic mice (CD1 background) were purchased from Jackson Laboratory and bred on the premises.

Adriamycin nephropathy, BIO treatment Adriamycin nephropathy was induced in five week-old male *NPHS2.iCreERT2;mT/mG* (n=31), *Pax2.rtTA;TetO.Cre;mT/mG* (n=32) and *RARE-lacZ* mice (n=12) by two successive retro-orbital injections of doxorubicin hydrochloride (Sigma-Aldrich, 18 mg/kg in PBS for *NPHS2.iCreERT2;mT/mG* and *Pax2.rtTA;TetO.Cre;mT/mG* mice and 14 mg/kg in PBS for *RARE-lacZ* mice). At least 4 healthy mice were included in each experiment. Urinary albumin and creatinine ratio was evaluated on spot urine using Albuwell M kit (Exocell, Philadelphia, PA, USA), and Creatinine Assay kit

(Cayman Chemical, Ann Arbor, MI, USA). At day 28, mice were sacrificed and kidneys collected as described above. BUN levels were measured by Reflotron System (Roche Diagnostics, Rotkreuz, Switzerland) on blood samples collected at sacrifice.

The glycogen synthase inhibitor 6-Bromo-Indirubin-3'-Oxime (BIO) was obtained from Merck (KGaA, Darmstadt, Germany). For BIO treatment, *NPHS2.iCreERT2;mT/mG* and *Pax2.rtTA;TetO.Cre;mT/mG* mice received daily i.p. injections of BIO (5 µmol/kg, 5 days/week) or its vehicle DMSO starting from day 7 after the second doxorubicin for 3 weeks. Urinary albumin and creatinine ratio was evaluated on spot urine as described above.

In an additional experiment, Adriamycin nephropathy was induced in n=8 *NPHS2.iCreERT2;mT/mG* mice as described above. Two weeks after the second doxorubicin administration, animals underwent a biopsy. After anesthetization by intraperitoneal injection of 250 mg/kg 2,2,2-tribromoethanol (Avertin, Sigma-Aldrich), the kidney was exteriorized and a small piece of tissue was cut off using small scissors. A sterile gauze saturated with tranexamic acid (Tranex, Lusofarmaco, Peschiera Borromeo, Italy) was applied to the wound, which was then cauterized. The remaining kidney was pushed back in the body cavity and the animal sutured. On day 28, mice were euthanized and kidneys were collected as described above.

Analysis of glomerular injury Sections of 5 µm from formalin fixed-paraffin embedded kidneys were stained with periodic acid-Schiff reagent (Carlo Erba, Milan, Italy). Fifty randomly selected glomeruli were assessed for glomerular damage. The percentage of glomeruli with presence of sclerosis was evaluated by two independent observers in at least 50 glomeruli of at least four sections.

Immunofluorescence and Confocal Microscopy The following antibodies were used: anti-NPHS2 (pAb, ab50339, Abcam, Cambridge, United Kingdom) anti-SYN (mAb, G1D4, Progen, Heidelberg, Germany), anti-nephrin (pAb C-17, sc-32259, Santa Cruz Biotechnology), anti-WT1 (pAb C-19, sc-192, Santa Cruz Biotechnology), Alexa-Fluor secondary antibodies were obtained from Molecular Probes (Life Technologies, Monza, Italy). For the *Pax2.rtTA;TetO.Cre;R26.Confetti* mice, the acquisition was set in the cyan, green, yellow, and red wavelengths using 405, 488, 514, and 543 nm wavelength excitation, respectively. NPHS2 and WT1 staining was performed using an Alexa Fluor 647 goat anti-rabbit secondary antibody excitated with the 633 nm wavelength laser line. SYN staining was performed using an Alexa Fluor

647 goat anti-mouse IgG1 secondary antibody excitated with the 633 nm wavelength laser line. Nuclei were counterstained with DAPI (Life Technologies), excitated with infrared laser at 689 nm.

Z-serie stacks were collected from a 30 µm kidney slice, 1 µm optical slice thickness, 12 z-sections collected at 1 µm intervals (Figure S2B).

To generate 3D image (Figure 4L) a Java based image processing software (available at http://rsb.info.nih.goc/ij, developed by Wayne Rasband, National Institutes of Health, Bethesda, MD) was used. Forty-one z-sections (from a total of 64 images) collected at 0.64 µm intervals from the same glomerulus were overlapped to generate 3D reconstruction.

Podocyte quantification In *NPHS2.iCreERT2;mT/mG* mice we quantified:

1) the percentage of podocyte loss (P_{loss}) = 100 – mean of n° of total podocytes (green/blue + red/blue cells) at day 28 in proteinuric mice/mean of n° of total podocytes in healthy mice x 100;

2) the percentage of newly generated podocytes (P_{new}) = mean of n° of regenerated podocytes (red/blue cells)/mean of n° of total podocytes at day 28 in proteinuric mice x 100 – mean of n° of regenerated podocytes (red/blue cells)/mean of n° of total podocytes in healthy x 100;

3) total percentage of lost podocytes (ablated podocytes, P_{abl}) = P_{loss} + P_{new}.

The percentage of regenerate podocytes over lost podocytes showed in Figure 2G, J and Figure 7D was thus = P_{new}/P_{abl} x 100.

Similar formulas were used in *Pax2.rtTA;TetO.Cre;mT/mG* mice, where podocytes was quantified as the number of SYN+ or WT1+ cells per glomerular section in 15 glomeruli of at least four sections for each mouse counted by two independent observers (Figures 5H and 7K).

Screening of small library compounds The Stem cell-focused small molecule library (Cayman Chemical, Ann Arbor, Michigan, USA) was screened. To this aim, hRPC obtained as previously described (Ronconi et al., 2009) were treated for 48 hours with DMEM-F12 supplemented with 10% FBS, 100 µM RA, in presence or absence of each molecule (1 µM). The expression of nephrin at both mRNA and protein levels was then evaluated.

Cell Cycle Analysis Cell cycle analysis was performed on cells treated for 72 hours with DMEM-F12 + 10% FBS in presence or absence of 1 µM BIO using DAPI (Life Technologies) following manufacturer's instructions. Cell were analyzed with the Modfit LT 3.0 software (Verity Software House Inc., Topsham, ME, USA).

Real-Time quantitative RT-PCR Total RNA was extracted using an RNeasy Microkit (Qiagen, Hilden, Germany) and retrotranscribed using TaqMan Reverse Transcription Reagents (Life Technologies). TaqMan RT-PCR was performed using commercially available Assay on Demand kits (Life Technologies): nephrin cat. n. Hs00190446-m1, $RAR\alpha$ cat. n. Hs00940446-m1, $RAR\beta$ cat. n. Hs00977140-m1, $RAR\gamma$ cat. n. Hs01559234-m1, $RXR\alpha$ cat. n. Hs00172565-m1, $RXR\beta$ cat. n. Hs00232774-m1 and $RXR\gamma$.cat. n. Hs00199455-m1. For luciferase quantification, the following primers and probes were used (Life Technologies): probe 5'-VIC-ACGCCGGTGAACTTCCCGCC-TAMRA-3'; forward 5'-CGCAGGTCTTCCCGACG-3'; reverse 5'-TTCCGTGCTCCAAAACAACA-3'.

hRPC infection pGF1-RARE, which expresses firefly luciferase reporter under the control of RARE, the control empty vectors pGF1-CMV, and pGF1-mCMV were obtained from System Biosciences (SBI, CA, USA). Lentiviral particles were produced by cotransfection of the lentiviral plasmid and the packaging vectors into Lenti-X 293T cells (Clontech, Mountain View, CA, USA).

hRPC were infected with virus-containing supernatant with a MOI of 30 as well as fresh endothelial cell growth medium-microvascular (EGM-MV) media, in the presence of 8 µg/ml of polybrene. Level of infection, assessed by measuring percentages of ZsGreen1+ cells by flow cytometry, ranged from 40% to 90% of cells. Infection levels were further verified by quantitative PCR of luciferase DNA. Infected hRPC were treated with 100 µM RA in presence or absence of 1 µM BIO for 24 hours and then luciferase activity was evaluated.

For luciferase assay a commercially available kit (Promega, Milan, Italy) was used. The reporter activity of the pGF1-RARE infected cells was measured on a GloMax® 96 Luminometer (Promega) and normalized to the cells infected with pGF1-mCMV.

β-Gal assay Kidneys of *RARE-lacZ* mice were incubated for 30 minutes in an ice-cold fixative (3% PFA, 0.2% glutaraldehyde, 2.5 mM EGTA, and 4 mM $MgCl_2$ in 0.5× PBS [pH, 7.6], all from Sigma-Aldrich) and

then for 30 minutes in PBS, then frozen. beta-Gal assay was performed as previously described (Peired et al., 2013). X-Gal staining was combined with immunohistochemistry for the expression of claudin-1 (pAb, 71-7800, Life Technologies). As secondary antibody we used a biotinylated goat anti-rabbit (Vector Laboratories, United Kingdom). Detection was carried out with Vectastain ABC kit (Vector Laboratories) and 3-amino-9-ethylcarbazole (Vector Laboratories) as peroxidase substrate. The number of β-Gal+/claudin1+ cells present in glomeruli of DMSO- and BIO-treated mice was evaluated by two independent observers in at least 50 glomeruli of at least four sections.
