# Supplementary figures and images for: Podocyte Regeneration Driven by Renal Progenitors Determines Glomerular Disease Remission and Can Be Pharmacologically Enhanced
Source: Stem Cell Reports. 2015 Jul 30;5(2):248–63. doi: 10.1016/j.stemcr.2015.07.003 (PMC4618832; doi:10.1016/j.stemcr.2015.07.003)

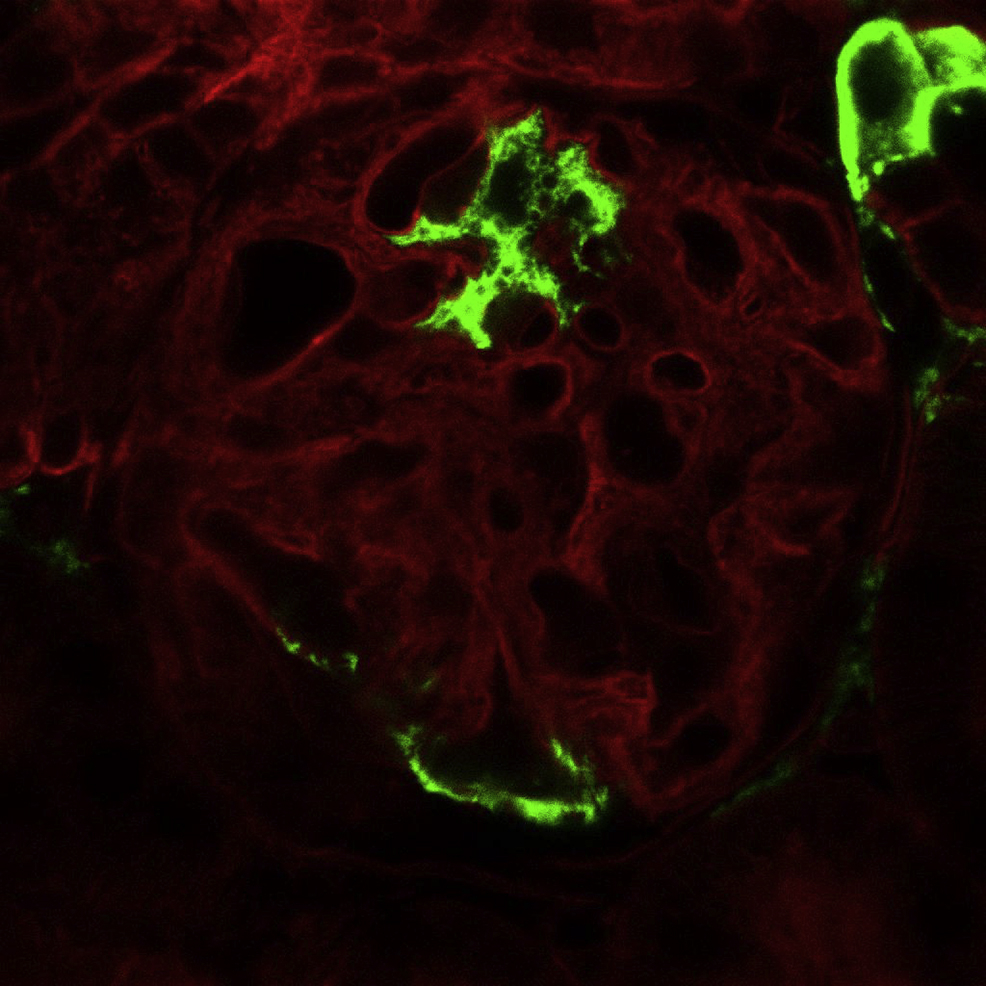

Supplement: Movie S1. PAX2+ Progenitors of the Bowman’s Capsule Generate New Podocytes, Related to Figure 4 [file mmc2.jpg]
